# Circulating proteins linked to apoptosis processes and fast development of end-stage kidney disease in diabetes

**DOI:** 10.1172/jci.insight.178373

**Published:** 2024-10-22

**Authors:** Katsuhito Ihara, Eiichiro Satake, Parker C. Wilson, Bozena Krolewski, Hiroki Kobayashi, Zaipul I. Md Dom, Joseph Ricca, Jonathan Wilson, Jonathan M. Dreyfuss, Monika A. Niewczas, Alessandro Doria, Robert G. Nelson, Marcus G. Pezzolesi, Benjamin D. Humphreys, Kevin Duffin, Andrzej S. Krolewski

**Affiliations:** 1 Research Division, Joslin Diabetes Center, Boston, Massachusetts, USA.; 2 Department of Medicine, Harvard Medical School, Boston, Massachusetts, USA.; 3 Department of Nephrology, Tokyo Medical and Dental University, Tokyo, Japan.; 4 Department of Pathology and Immunology, Washington University in St. Louis, St. Louis, Missouri, USA.; 5 Division of Diagnostic Innovation, Department of Pathology and Laboratory Medicine, University of Pennsylvania, Philadelphia, Pennsylvania, USA.; 6 Division of Nephrology, Hypertension, and Endocrinology, Nihon University School of Medicine, Tokyo, Japan.; 7 Diabetes and Complication Department, Lilly Research Laboratories, Eli Lilly and Company, Indianapolis, Indiana, USA.; 8 Division of Nephrology and Hypertension, Department of Internal Medicine, University of Utah School of Medicine, Salt Lake City, Utah, USA.; 9 Division of Nephrology, Department of Medicine, Washington University in St. Louis, St. Louis, Missouri, USA.

**Keywords:** Nephrology, Apoptosis, Diabetes

## Abstract

Many circulating proteins are associated with risk of ESKD, but their source and the biological pathways/disease processes they represent are unclear. Using OLINK proteomics platform, concentrations of 455 proteins were measured in plasma specimens obtained at baseline from 399 individuals with diabetes. Elevated concentrations of 46 circulating proteins were associated (*P* < 1 × 10^–5^) with development of ESKD (*n* = 143) during 7–15 years of follow-up. Twenty of these proteins enriched apoptosis/TNF receptor signaling pathways. A subset of 20 proteins (5–7 proteins), summarized as an apoptosis score, together with clinical variables accurately predicted risk of ESKD. Expression of genes encoding the 46 proteins in peripheral WBCs showed no difference between cells from individuals who did or did not develop ESKD. In contrast, plasma concentration of many of the 46 proteins differed by this outcome. In single-nucleus RNA-Seq analysis of kidney biopsies, the majority of genes encoding for the 20 apoptosis/TNF receptor proteins were overexpressed in injured versus healthy proximal tubule cells. Expression of these 20 genes also correlated with the overall index of apoptosis in these cells. Elevated levels of circulating proteins flagging apoptotic processes/TNF receptor signaling pathways — and likely originating from kidney cells, including injured/apoptotic proximal tubular cells — preceded the development of ESKD.

## Abstract 2

Elevated levels of multiple circulating proteins flagging apoptotic processes/TNF receptors signaling pathways in damaged proximal tubule cells preceded fast progression to ESKD in individuals with diabetes.

## Graphical abstract

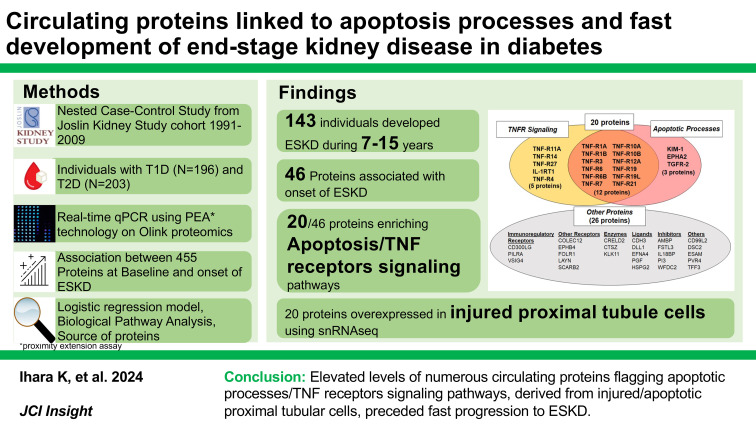

## Introduction

Diabetic kidney disease (DKD) is responsible for more than 40% of individuals with end-stage kidney disease (ESKD) in the US (1). A large proportion of these individuals had ESKD resulting from fast kidney function decline (2, 3). Despite significant research efforts, the specific disease processes underlying fast development of ESKD are still unclear (4). Over the last decade, intensive research has sought to find biomarkers associated with ESKD risk so that this disease process can be elucidated and tools to diagnose individuals at risk can be developed. Recently, the search for new biomarkers of DKD has been accelerated by the development and application of high throughput proteomics platforms. These platforms enable the simultaneous quantification of hundreds to thousands of circulating proteins that can be examined as candidate biomarkers for ESKD risk (5, 6).

Previously, using an aptamer-based SOMAscan proteomics platform (5), we performed several studies among individuals with diabetes in multiple cohorts and identified 40 circulating proteins robustly associated with ESKD risk (7–11). Several other studies, conducted in general populations that used the SOMAscan platform, identified many, frequently the same as ours circulating proteins being associated with progressive kidney function decline (12–14). Despite these associations, little progress has been made in defining the biological pathways/disease processes these proteins represent and in determining the sources and mechanisms of their elevated concentrations in circulation.

In the present global-untargeted proteomic study, we used 2 nested case-controls studies; one included individuals with type 1 diabetes (T1D) and the other with T2D. In both studies, we examined the relation between baseline concentration of 455 plasma proteins and risk of development of ESKD during 7–15 years of follow-up. Our primary goal was to find circulating proteins with abnormal concentrations that preceded fast progressive kidney function decline so mechanisms/pathways associated with initiation/maintenance of disease processes leading to ESKD could be elucidated. We used statistical modeling to demonstrate that subsets of proteins enriching the apoptosis/TNF receptor signaling pathways were the most important for prediction of ESKD risk.

The OLINK Proseek Multiplex proteomics platform that measures proteins by quantitative PCR (qPCR) using proximity extension assay (PEA) technology was used (6). This technology is different from the aptamer-based technology applied in the SOMAscan platform, which was used by us and others in the previous studies. Although many of the same proteins were measured by both platforms, OLINK measurements are more specific and more precise than the SOMAscan measurements (15). In addition, new proteins are quantified by the OLINK platform. Therefore, using the OLINK platform, this study aimed to search for new circulating ESKD risk–associated proteins not measured or not detected by SOMAscan platform. Auxiliary studies were performed to search for possible sources/origins of elevated circulating proteins associated with ESKD risk.

## Results

### Characteristics of study groups

**Figure 1:**
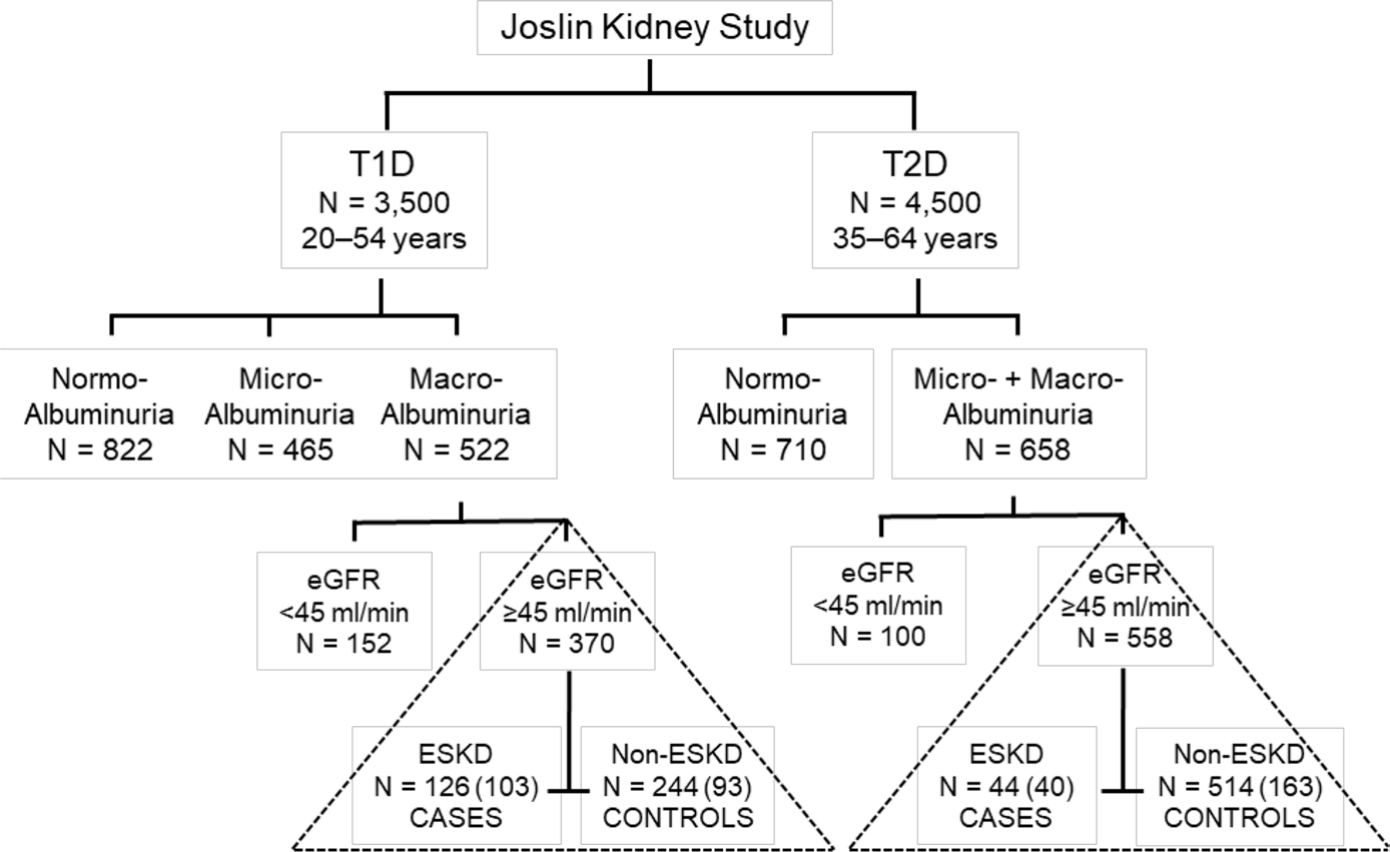
Selection of individuals for the 2 nested case-control studies. The Joslin Kidney Study (JKS) enrolled 370 individuals with T1D with baseline Macro-albuminuria and eGFR ≥ 45 mL/min/1.73 m^2^. During 7–15 years of follow-up, 126 of these individuals developed end-stage kidney disease (ESKD) (cases) and 244 did not (controls). For the current study, 103 cases were available and 93 controls were selected randomly. The JKS enrolled 558 individuals with T2D with baseline Micro- and Macro-albuminuria and eGFR ≥ 45 mL/min/1.73 m^2^. During 7–15 years of follow-up, 44 of these individuals developed ESKD and 514 did not. For the current study, 40 cases were available and 163 controls were selected randomly.

This investigation comprises 2 nested case-control studies. Participants were selected from among individuals who were enrolled into the Joslin Kidney Study (JKS) and were followed for 7–15 years. The outline of the JKS and selection of individuals for this investigation are illustrated in Figure 1 . Financial constraints limited the OLINK measurements to just 400 individuals, and we sought to include approximately equal numbers of persons with T1D and T2D for this study. For the T1D Macro-albuminuria study (Discovery study), we selected 103 individuals who progressed to ESKD during follow-up (cases) and 93 randomly selected individuals from among those who did not progress to ESKD (controls). Similarly, for the T2D Albuminuria (included Macro- and Micro-) study (Replication study), we selected 40 individuals who progressed to ESKD during follow-up (cases) and 163 individuals randomly selected from among those who did not progress to ESKD. Ninety percent of participants in the JKS are White.

Table 1 presents characteristics for cases and controls in each study at baseline and during follow-up. Individuals with T1D were younger and had longer duration of diabetes than those with T2D. At baseline, cases had higher HbA_1c_ and urinary albumin/creatinine ratio (ACR) and lower eGFR in comparison with controls in both studies. During follow-up, median eGFR loss was approximately 10 mL/min/1.73 m^2^/year in cases in both studies. This indicates that cases with ESKD in T1D and T2D had similar fast kidney function decline. In both studies, there were only a few deaths unrelated to ESKD during follow-up.

### Search for proteins associated with fast development of ESKD

**Figure 2:**
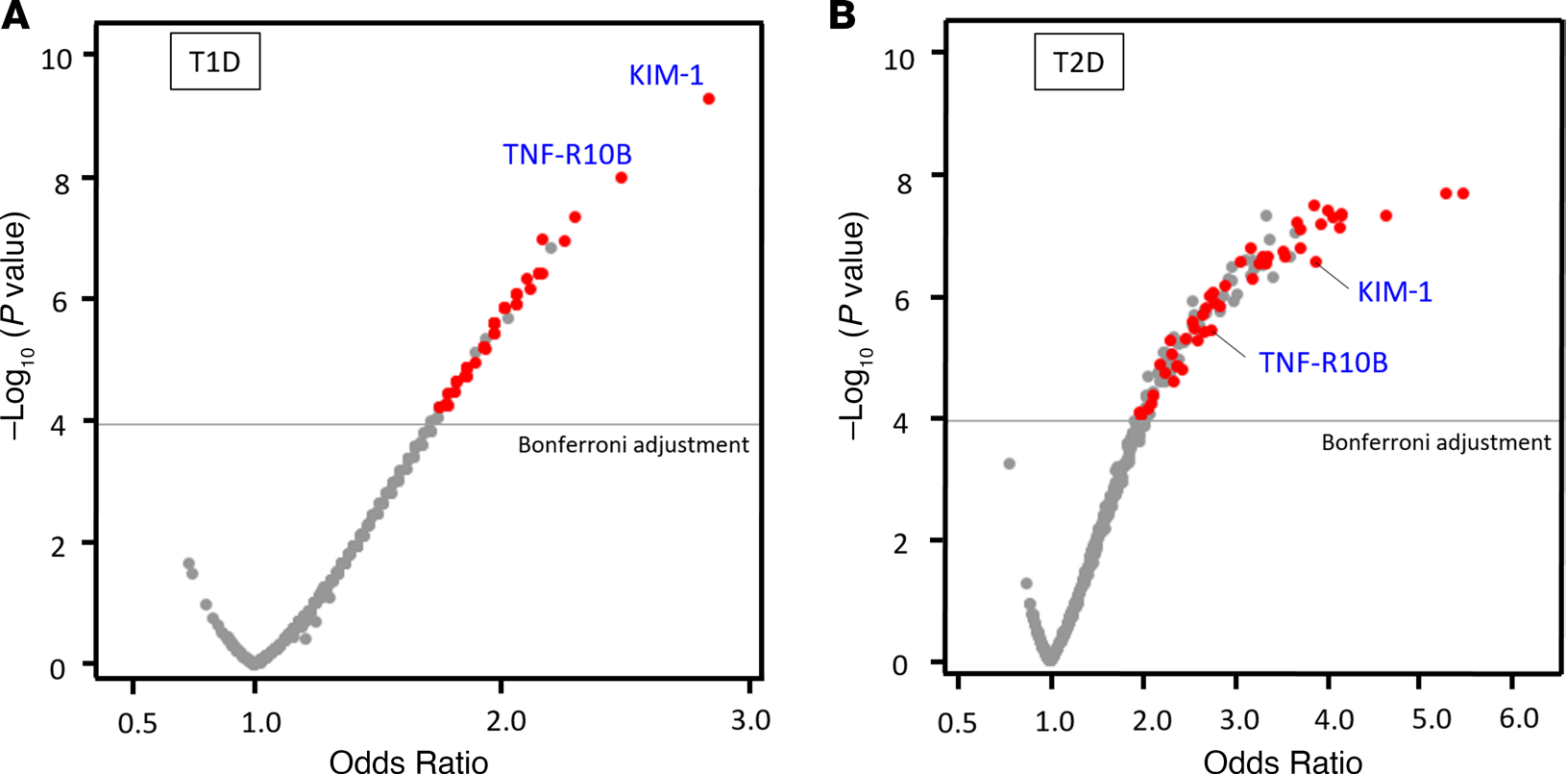
Associations between 455 proteins and development of ESKD. Volcano plots of OR for the development of ESKD for 455 proteins in T1D study (**A**) and in T2D study (**B**). Using Bonferroni-adjusted *P* values, 46 proteins were statistically significant for association with fast development of ESKD in both T1D and T2D studies. These proteins are shown in red.

Baseline plasma specimens — i.e., obtained at the beginning of the follow-up — from both studies were used to quantify 455 proteins using OLINK Proseek platform. In the T1D Discovery study, concentrations of 50 proteins were associated with risk of fast progression to ESKD as indicated by significant odds ratio (OR). Distribution of ORs according to *P* values in this study is shown as a volcano plot in Figure 2A. In the T2D Replication study, 43 proteins were confirmed (indicated as red dots in Figure 2B). Three additional proteins (TNF-R4, TNF-R6, and TNF-R21) were included in the further analyses. These proteins were associated with ESKD risk in our previous studies (7, 16) and in the current study; however, after Bonferroni adjustment, they remained statistically significant only in the T2D study. In total, 46 circulating proteins were analyzed, and they were referred to as ESKD risk–associated proteins. Our study examined the concentrations of 46 proteins between males and females, and similar findings are reported for both sexes.

Comparing the results between T1D and T2D, ORs for many of these proteins seem to be higher in T2D than in the T1D study. However, heterogeneity testing showed that only adjusted ORs for EPHB4 showed evidence of heterogeneity of results (Supplemental Table 1; supplemental material available online with this article; https://doi.org/10.1172/jci.insight.178373DS). Therefore, the results for T1D and T2D were combined for further analyses. The names of the 46 ESKD risk–associated proteins and OR for each of them in the combined studies are shown in Table 2.

**Figure 3:**
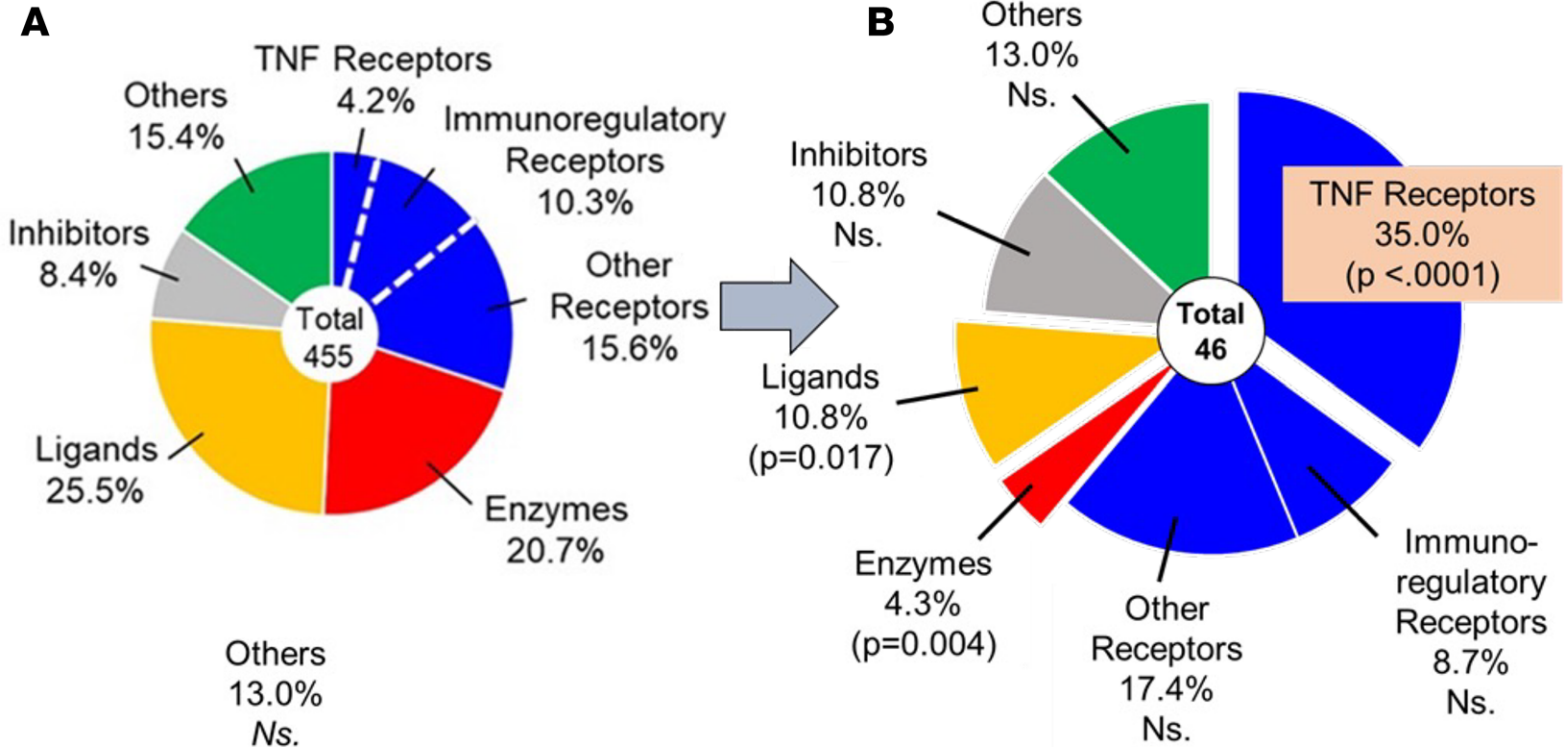
Distribution of proteins by functional categories in all examined proteins and proteins associated with development of ESKD. (**A**) In total, 455 proteins on the OLINK proteomics platform were used for the analysis. They were categorized based on protein function from literatures sources: TNF receptors, immunoregulatory receptors, other receptors, enzymes, ligands, inhibitors, and others. (**B**) Similarly, the 46 ESKD risk–associated proteins were categorized based on protein function as above. Proportion of proteins between the 2 sets were compared using a chi-square test.

Proportions of proteins according to functional categories among the 455 proteins measured on the OLINK platform and among the 46 ESKD risk–associated proteins were compared, and the results are presented in Figure 3 . The proportion of circulating proteins associated with ESKD risk varied among the categories. We found a striking excess of ESKD risk–associated proteins in the category of TNF receptors; 35.0% observed versus 4.2% expected (*P* < 0.0001). Other categories were not enriched with ESKD risk–associated proteins, and there were deficiencies of ESKD risk–associated proteins in categories of enzymes — 4.3% versus 20.7% expected (*P* = 0.004) — and ligands — 10.8% versus 25.5% (*P* = 0.017).

### Biological pathways/terms enriched by ESKD risk–associated proteins

**Figure 4:**
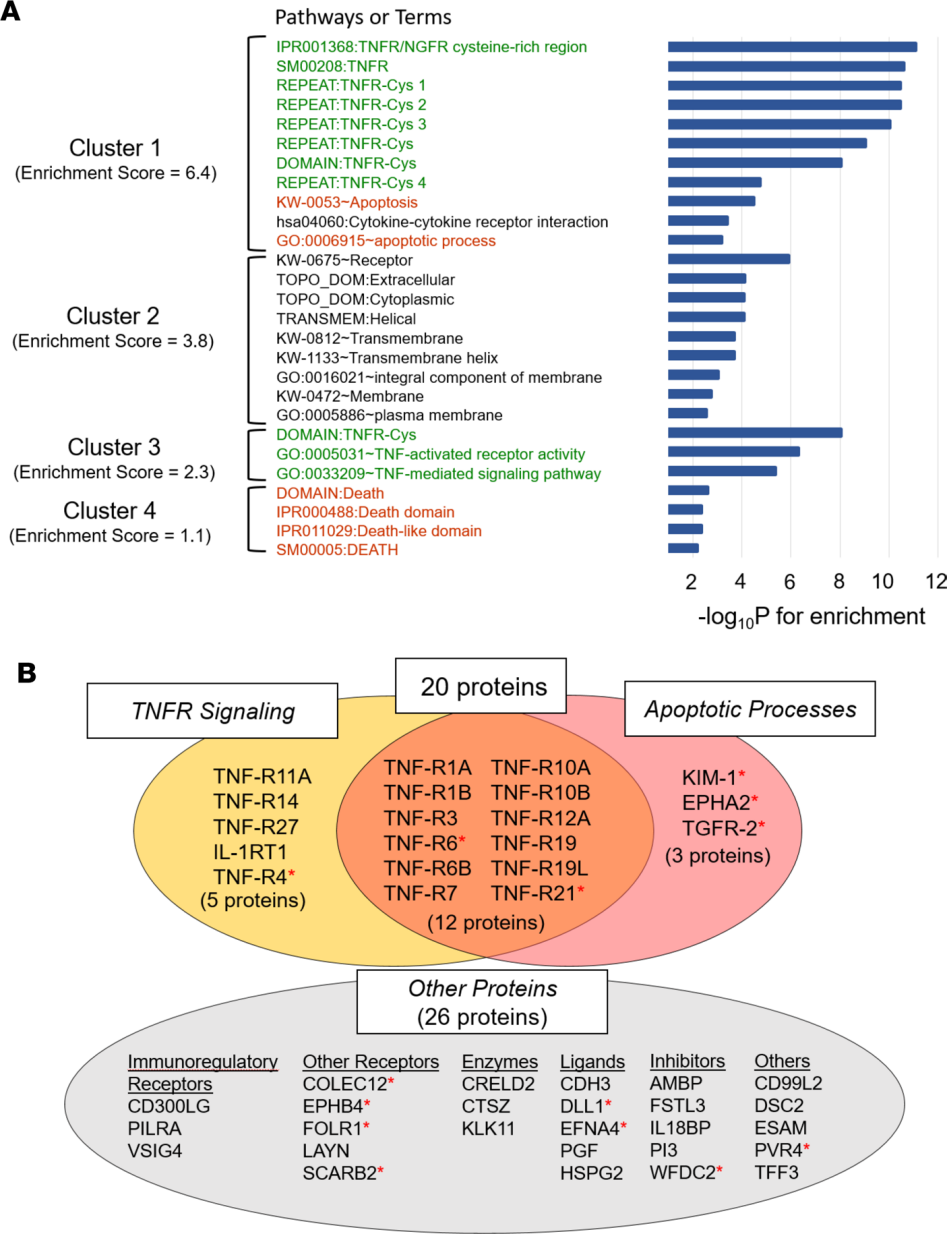
Results of pathway analysis of 46 ESKD risk–associated proteins. (**A**) Clusters of enriched pathways by 46 ESKD risk–associated proteins are shown. The functional annotation clustering in DAVID software (version 6.8) was applied for pathway enrichment analysis using the DAVID databases (e.g., GO, KEGG, REACTOME pathways and UniprotKB keywords). The results were obtained using 455 proteins measured on 5 OLINK panels. Similar results were obtained when all 1,012 proteins included in 11 OLINK panels were used as background (data not shown). The geometric mean (in –log scale) of the Expression Analysis Systemic Explorer (EASE) scores (modified Fisher exact *P* values) were used to rank their biological significance. Four clusters of pathways or terms with EAS E scores of *P* < 0.01 are shown in the figure as statistically significant. More detailed results are shown in Supplemental Table 2. Result of similar analysis for SOMAscan data are shown in Supplemental Figure 3. (**B**) Venn diagrams of 46 ESKD risk–associated proteins grouped according to enriched pathways are shown. Twenty proteins referred to as apoptosis/TNF receptor proteins were enriching pathways in clusters 1, 3, and 4 in **A**. Twenty-six other proteins did not enrich any known pathway. However, 8 of these proteins and all 20 apoptosis/TNF receptor proteins were enriching pathways included in cluster 2 referred to as receptors and membrane proteins. Asterisks indicate proteins found to be associated with sickle cell related kidney disease (18).

To explore the possible role of the 46 ESKD risk–associated proteins in the disease process that underlies fast development of ESKD, we examined which biological pathways/terms were enriched with the ESKD risk–associated proteins. We performed pathway analysis using the Database for Annotation, Visualization, and Integrated Discovery (DAVID) Bioinformatics Resources 6.8 (https://david.ncifcrf.gov/) (17). Figure 4A and Supplemental Table 2 show the results of such analysis. Four clusters of pathways or terms showed highly statistically significant enrichment. The first cluster had the highest score of enrichment (score, 6.4) and comprised apoptotic processes and TNF receptor signaling pathways. The second cluster comprised receptors and membrane proteins as biological terms with score of enrichment (score, 3.8). The third and fourth cluster comprised proteins enriched in apoptotic processes and TNF receptor signaling and had moderate scores of enrichments (scores, 2.3 and 1.1, respectively). No other cluster was identified as statistically significant in the pathway analysis. Inspection of proteins that contributed to enrichment in clusters 1, 3, and 4 revealed 20 proteins. These proteins are referred to as apoptosis/TNF receptor proteins. Figure 4B shows that, among these proteins, 12 were common for apoptosis processes and TNF receptor signaling, 3 were specific for apoptosis, and 5 were specific for TNF receptor signaling. Almost all these proteins were also enriching cluster 2 that comprised terms receptors or membranes. It is of interest to note that circulating proteins found to be associated with sickle cell–related kidney disease (Figure 4B) and measured by OLINK platform did not enrich the apoptosis/TNF receptor signaling pathways (18).

### Sources/origins of ESKD risk–associated proteins

#### Expression of genes encoding ESKD risk–associated proteins in peripheral WBC.

**Figure 5:**
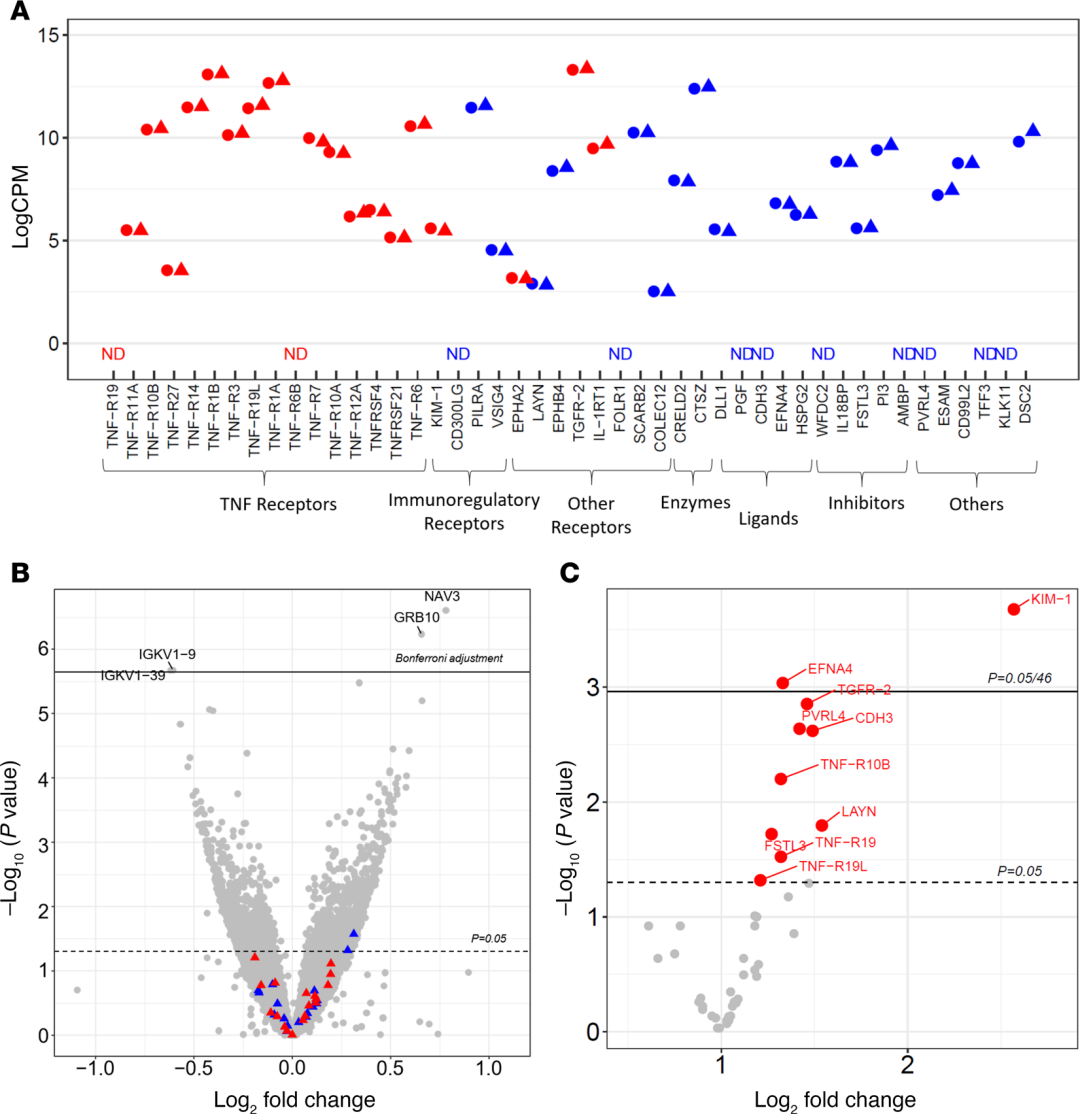
Comparison of expression of 46 genes encoding ESKD risk–associated proteins in circulating WBC and concentration of these proteins in plasma obtained at baseline in individuals who did develop (16 cases) and did not develop ESKD (55 controls) during 7–15 years of follow-up. Clinical characteristics of cases and controls are shown in Supplemental Table 3. (**A**) Comparison between cases (triangles) and controls (circles) of mean expression levels of mRNAs in WBC of genes encoding for the 46 ESKD risk–associated proteins. Genes encoding 20 apoptosis/TNF receptor proteins are indicated in red, and genes encoding 26 other proteins are indicated in blue. ND, not detected. (**B**) Volcano plot of log_10_ of *P* value against log_2_ of fold change of expression of 22,121 genes detected in WBC in cases over controls. No gene was found to be differentially expressed in WBC among these 2 groups following multiple testing correction (Bonferroni-adjusted *P* < 2.26 × 10^–6^). Red triangles indicate genes encoding for apoptosis/TNF receptor proteins. Blue triangles indicate genes encoding for other proteins. (**C**) Volcano plot of log_10_ *P* value against fold change of 46 plasma concentration of ESKD risk–associated proteins in cases over controls. Out of 46 proteins, 10 were significantly upregulated in cases (in red).

A genomic study was conducted in which mRNA was extracted from peripheral WBC collected into PAX gene tubes and sequenced. PAX gene tubes were collected at baseline from 23 T1D and 48 T2D individuals (total, 71) included in this study. During 7–15 years of follow-up, 16 of these individuals developed ESKD and 55 did not (Supplemental Table 3). Figure 5A shows a comparison of the expression of 46 genes encoding the ESKD risk–associated proteins by outcome. A threshold of 6 log counts per million (logCPM) was used to determine low expression. Eleven genes of interest were not expressed in WBC, 10 had low expression, and 25 had moderate or high expression. Strikingly, expression of these genes was identical in those who progressed and those who did not progress to ESKD. As shown in Figure 5B, only a few other genes out of 22,121 were found to be differentially expressed between the 2 groups after adjusting for multiple testing correction (Bonferroni-adjusted, *P* < 2.26 × 10^–6^). The negative genomic results contrasted with the proteomics findings (Figure 5C). All 46 proteins were detected and measured in baseline plasma. For the 11 genes not expressed in WBC, the corresponding proteins had moderate plasma concentrations, and for 3 of them, *TNFR19*, *CDH3*, and *PVRL4*, these concentrations were significantly higher in individuals who developed ESKD than in those who did not. Among the 35 remaining genes expressed in WBC equally in cases and controls, baseline plasma concentrations of 7 corresponding proteins were significantly higher (higher fold change [FC]) in individuals who developed ESKD than in those who did not (*TNFR10B*, *TNFR19L*, *KIM1*, *LAYN*, *TGFR2*, *FSTL3*, and *EFNA4*) (Figure 5C). Additionally, FC for 3 proteins were higher (*TNFR27*, *PI3*, and *TFF3*) and 4 were lower (*TNFR6*, *EPHB4*, *AMBP*, and *CD99L*), but the differences were not statistically significant. Plasma concentrations for 21 remaining proteins were similar in cases and controls (FC around 1.0).

#### Expression of genes encoding ESKD risk–associated proteins in kidney cells.

In a previous study, single nucleus sequencing of kidney cortex obtained from individuals with DKD identified a subset of injured proximal tubule cells that had a distinct transcriptional and chromatin accessibility profile (19). These injured proximal tubule cells often express *VCAM1* and variably express other injury-associated markers like *CD24* and *CD133*. In this study, we reanalyzed the previously published data (20) to examine expression of the 46 genes encoding the circulating ESKD risk–associated proteins in proximal tubules. We compared the differential expression of these genes and differential accessible chromatin regions in the loci for these genes between control proximal tubule and injured proximal tubule (PT_VCAM1).

**Figure 6:**
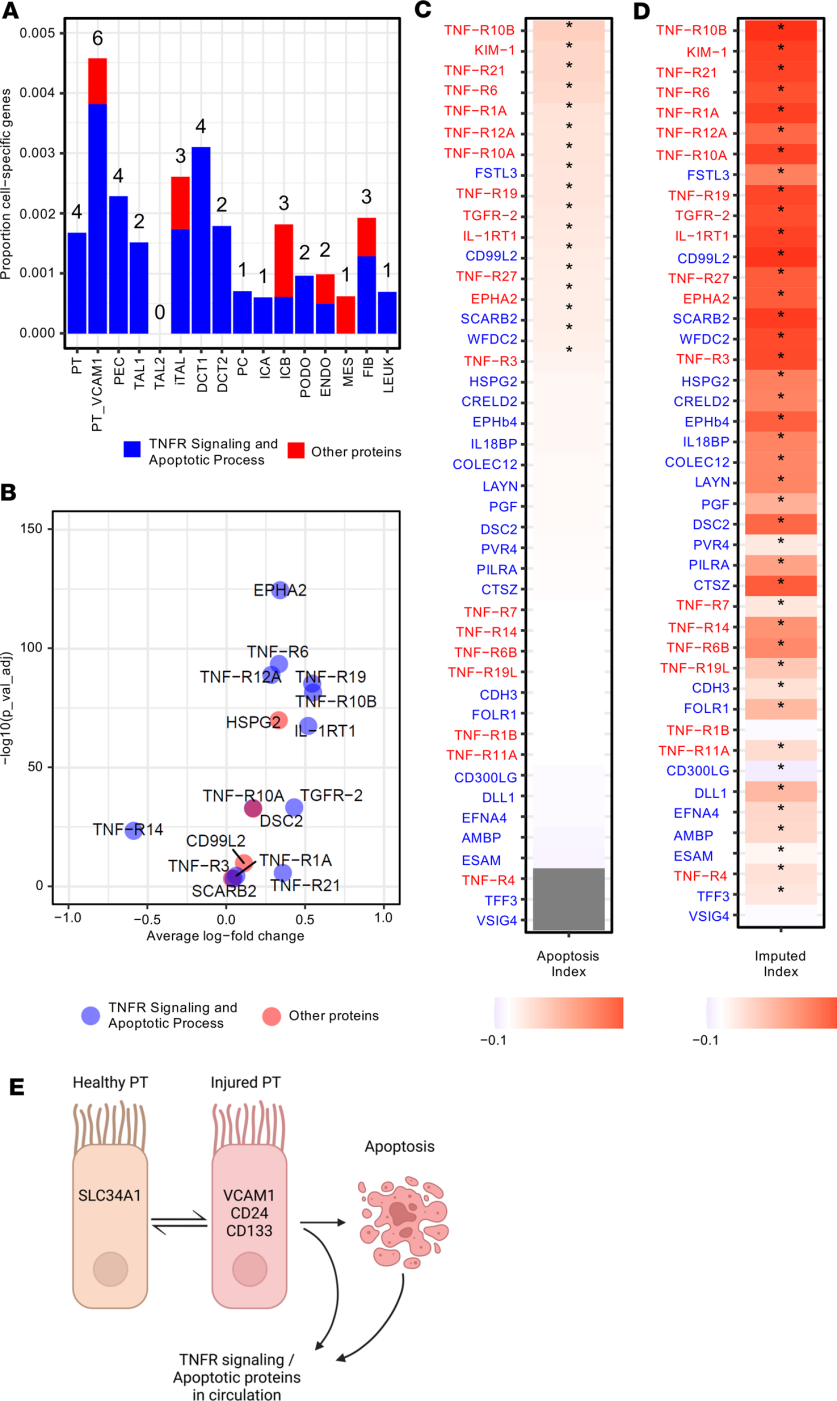
snRNA-Seq analysis and enrichment of apoptosis pathways and TNF receptor proteins in injured (PT_VCAM1) and control proximal tubule cells. Reanalyzed data from previous publication (19–21). The gene names for the proteins in the Figure are listed in Supplemental Table 1. (**A**) Cell-specific expression of gene encoding for 20 apoptosis/TNF receptor proteins and 26 other proteins among kidney cell types. Cell-specific genes were computed using the Seurat FindMarkers function comparing the indicated cell type to all other cell types using a Wilcoxon rank sum test with an adjusted *P* < 0.05 threshold. The intersection between cell-specific genes and the 20 apoptosis/TNF receptor proteins and 26 other proteins is displayed as a bar plot for each cell type. (**B**) Differential expression of genes encoding for 46 circulating ESKD risk–associated proteins in PT_VCAM1 versus control proximal tubule cells is shown. The *y* axis corresponds to a Benjamini-Hochberg adjusted *P* value and only 14 genes that meet the adjusted *P* value threshold (*P* < 0.05) are displayed. Blue circles indicate 11 apoptosis/TNF receptor proteins, and red circle indicate 3 other proteins. For this analysis, we analyzed 9,901 proximal tubule cells and 1,577 PT_VCAM1 cells. (**C**) Pearson correlation between an aggregate measure of Hallmark 205 apoptosis genes (apoptosis index) and genes encoding the 46 circulating ESKD risk–associated proteins was computed for the PT_VCAM1 cell state. Asterisks indicate genes that are significantly correlated with apoptosis index using an unadjusted *P* < 0.05. Genes that are filled gray were not detected in the snRNA-Seq dataset in the PT_VCAM1 cell state. Names in red indicate apoptosis/TNF receptor proteins. (**D**) Pearson correlation between an aggregate measure of hallmark 205 apoptosis genes (apoptosis index) and genes encoding the 46 circulating ESKD risk–associated proteins following imputation. See Supplemental Figures 1 and 2. (**E**) Escape hypothesis. Healthy proximal tubules express markers like SLC34A1 that mediate normal homeostatic functions. When proximal tubules are injured, they begin to express markers like VCAM1, CD24, and CD133 (21). The injured cells overexpressed genes encoding for the apoptosis/TNF receptor proteins, which are released into circulation. A subset of injured proximal tubule cells ultimately progress to apoptosis.

PT_VCAM1 expressed the greatest number of cell-specific genes among our list of 46 circulating proteins (Figure 6A). Other cell types that also expressed a significant number of these genes included the proximal tubule, injured thick ascending limb (iTAL) and distal convoluted tubule. The PT_VCAM1 cells showed increased expression of 14 genes of the 46 compared with control proximal tubule by single-nucleus RNA-Seq (snRNA-Seq) (Figure 6B). Importantly, 11 of these genes encode apoptosis/TNF receptor proteins. When expression of these genes was correlated with expression of 205 apoptosis pathway genes expressed as an aggregate apoptosis index, 17 of the 46 genes showed statistically significant positive correlation in the PT_VCAM1 injured cell state (Figure 6C). Thirteen of these genes encoded for apoptosis/TNF receptor proteins. To address sparsity of snRNA-Seq data, we performed the correlation after imputation of missing genes and showed that the vast majority of the 46 genes had a significant positive correlation (Figure 6D). To exclude the possibility that the positive correlation was due to chance alone, we used a linear regression model to estimate the effect size of all genes on increased apoptosis index in the imputed dataset. The estimated effect size of apoptosis/TNF receptor–related genes was significantly higher than the remaining genes in the genome (Supplemental Figure 1). PT_VCAM1 cells also showed increased accessibility of chromatin regions in the loci of 7 of these genes (*TNFR10A*, *TNFR10B*, *TNFR14*, *TNFR19*, *EPHA2*, *KIM1*, and *IL1RT1*). The regions were in promoters and/or introns of these genes (Supplemental Figure 2). All the above findings prompted us to hypothesize that, since injured proximal tubule cells had increased transcription of the apoptosis/TNF receptor–related genes, these cells might be the source of elevated levels of apoptosis/TNF receptor proteins in circulation in individuals with diabetes who are at risk of fast development of ESKD (see Figure 6E).

### Importance of apoptosis/TNF receptor proteins for prediction of ESKD risk

In the first stage, we used the 20 apoptosis/TNF receptor proteins to build the prediction model for ESKD risk. Performing multiple least absolute shrinkage and selection operator (LASSO) analyses, we found 3 combinations of these proteins, which had similar efficiency to predict ESKD risk (Supplemental Table 4). One of these sets, referred to as Model #1, is shown in Table 2 and includes 5 proteins: KIM-1, TNF-R27, IL-1RT1, TNF-R11A, and TNF-R6B. Based on their β coefficients, we developed the ESKD risk score, which we refer to as their apoptosis score. The OR for ESKD risk/quartile changes of the apoptosis score was 2.83 (95% CI, 2.23–3.59) without clinical variables and increased accordingly to 3.17 (95% CI, 2.18–4.62) when combined with clinical variables such as eGFR, ACR, and HbA_1c_.

In the second stage, in addition to 20 apoptosis/TNF receptor proteins, we examined contribution to prediction of ESKD risk of the 26 other ESKD risk–associated circulating proteins. LASSO regression analysis using all 46 ESKD risk–associated proteins identified 5 proteins from Model #1 and 5 proteins selected from among the other 26 proteins (Table 3). It is important to note that other proteins did not replace the apoptosis/TNF receptor proteins in this global model, but their effects were only diminished to make room for other proteins (Table 3). Importantly, a risk score created out of these 10 proteins did not provide better discrimination of ESKD risk than Model #1. Table 3 shows also inferior prognostic performance of Model #3, which included only the 3 currently used biomarkers: KIM-1, TNF-R1A, and TNF-R1B. All these models had significant additive prognostic values measured as c statistic over the clinical model (Table 3).

**Figure 7:**
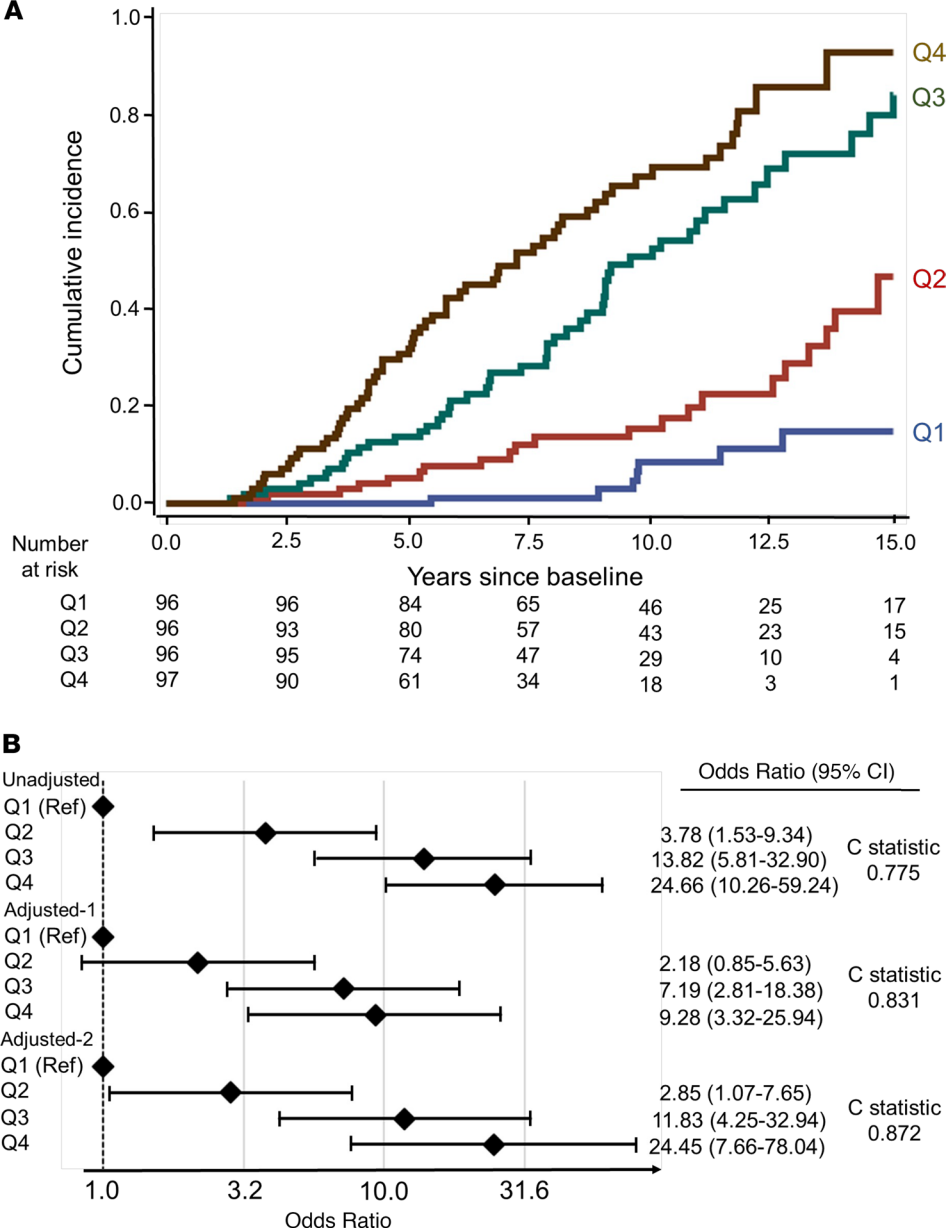
Risk of ESKD according to apoptosis score determined at baseline examination. (**A**) Cumulative incidence of ESKD during 15 years of follow-up according to quartiles of baseline apoptosis score. (**B**) ORs for 15-year risk of ESKD according to baseline quartiles of apoptosis score (score regarded as a categorical variable) without and with adjustment for relevant covariates: adjusted-1 (eGFR, ACR, HbA_1c_, and study indicator) and adjusted-2 (eGFR, ACR, HbA_1c_, age, systolic blood pressure, and study indicator). The ORs are represented on log_10_ scale.

To illustrate graphically the effect of the apoptosis score on the development of ESKD during 15 years of follow-up, we examined cumulative incidence of ESKD according to quartile of the score. As shown in Figure 7A, cumulative incidence of ESKD increased dramatically with increased quartiles of the score. Importantly, the adjustment for clinical variables did not change the very strong association between apoptosis score and risk of ESKD (Figure 7B).

### Comparison of OLINK findings with SOMAscan findings

In our previous studies, as well as in studies by others, only SOMAscan platform was used. In our studies, we examined multiple independent cohorts of individuals with T1D and T2D with DKD, and 40 circulating proteins were found as strong predictors of ESKD risk (10). The current study used the OLINK platform with 455 proteins and focused on individuals with T1D and T2D who had normal or only moderately impaired kidney function at baseline. Forty-six circulating proteins were found as strong predictors of development of ESKD during 7–15 years of follow-up. To compare the SOMAscan results versus the findings obtained using OLINK platform, 2 different approaches were used.

**Figure 8:**
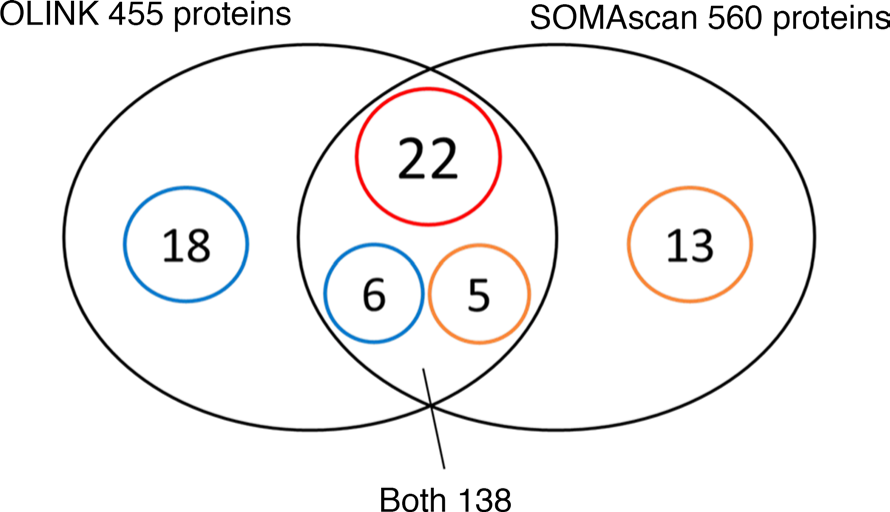
Venn diagram of overlapping proteins measured by OLINK and SOMAscan platforms. The small circles feature numbers of ESKD risk–associated proteins. Among 138 proteins measured by both platforms, there were 33 ESKD risk–associated proteins detected by 1 or both platforms. See more details about these proteins in Figure 9 below. Among proteins measured only on OLINK platform, 18 proteins were associated with ESKD risk. Among proteins measured only on SOMAscan platform, 13 proteins were associated with ESKD risk. Previously published results for SOMAscan measurements were used for comparisons (10).

**Figure 9:**
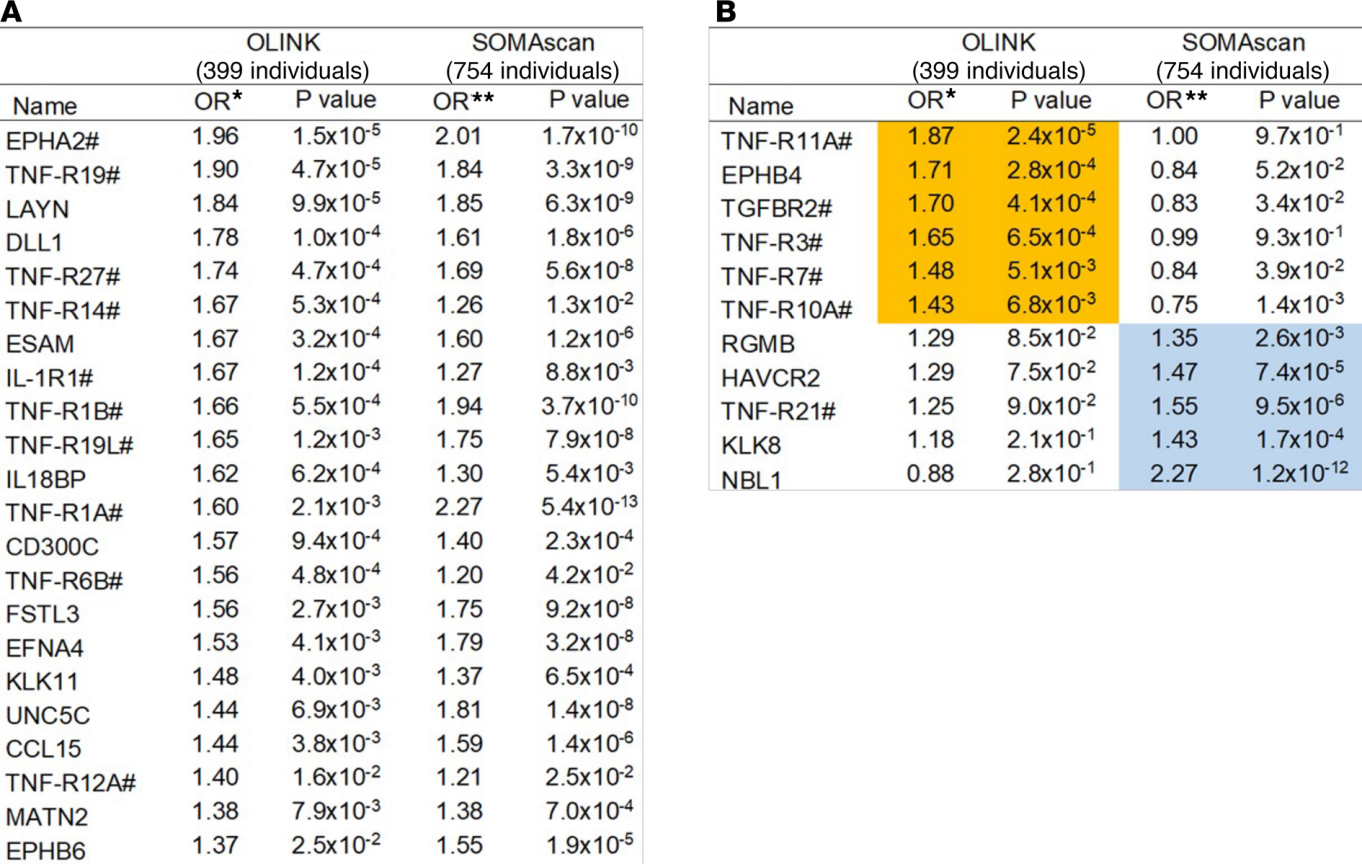
Names of ESKD risk–associated circulating proteins found using OLINK and SOMAscan proteomics platforms. (**A**) Proteins measured and associated with ESKD on both platforms. (**B**) Proteins measured on both platforms but associated with ESKD only on one platform. Asterisk indicates models adjusted for eGFR, ACR, HbA_1c_, and 2 study indicator. ORs for ESKD risk within 15 years are presented per 1 quartile increase in protein and ACR levels. Results obtained in the current study. Double asterisk indicates models adjusted for eGFR, log_2_ACR, HbA_1c_, and cohort indicator. ORs for onset of ESKD within 10 years are presented per 1 quartile increase in protein levels. Reanalyzed results from our previous publications are shown (10). ^#^Circulating proteins enriching apoptosis/TNF receptor signaling pathways. Proteins with orange showed strong association with ESKD risk only for OLINK measurements. Proteins with blue showed association with ESKD risk only for SOMAscan measurements.

In the first approach, individual proteins (Figure 8) were compared. Out of 455 proteins examined using OLINK platform and 560 proteins examined using SOMAscan, 138 proteins were measured by both platforms and 33 were associated with ESKD risk at least on one of the platforms. Out of these 33 proteins, 22 showed statistically significant association with ESKD risk using both platforms. See Figure 9A for the names of these proteins. In Figure 9B, there are names of proteins that showed association with ESKD risk but only on one of the platforms. There are 6 proteins, including 4 TNF receptors that showed strong association with ESKD risk, only for OLINK measurements. Five other proteins showed association with ESKD risk only for SOMAscan measurements. There were 18 circulating proteins associated with ESKD risk that were measured on OLINK only and 13 circulating proteins associated with ESKD risk that were measured on SOMAscan only. In total, of 877 proteins measured on one or both platforms, 64 circulating proteins were found to be associated with ESKD risk.

In the second approach, we compared results of pathway analysis for 46 ESKD risk–associated proteins detected by OLINK as shown in Figure 4A with similar analysis performed for 40 ESKD risk–associated proteins detected by SOMAscan (Supplemental Figure 3). The latter analysis showed similar results to the first one, although with different ranking. The top cluster for the 40 SOMAscan proteins comprised terms receptors and membranes. The second cluster comprised TNF receptor signaling pathways, and the third cluster comprised apoptosis terms. Overall, the enrichment scores and statistical significances for the pathways and terms in the 3 clusters in SOMAscan proteins were weaker than the results of the analysis for the 46 proteins measured on OLINK. The less-significant results obtained by analyzing the SOMAscan data resulted most likely from an inability of this platform to detect a number of apoptosis/TNF receptor proteins (Figure 9B). It is important to emphasize that no other individual pathways or cluster of pathways was statistically significantly enriched when the OLINK or SOMAscan findings were analyzed.

## Discussion

Many circulating proteins are associated with ESKD risk; however, it is still unknown what biological pathways/disease processes they represent. This study used the OLINK proteomics platform, which provides better measurements of circulating proteins than the SOMAscan platform, to search for such pathways in individuals with T1D and T2D with DKD. These individuals were selected to have normal or only slightly impaired kidney function at baseline, so our proteomic study identified initiators/early disease processes of kidney function decline that underlie the development of ESKD, and the results were not confounded by impaired kidney function. We also considered as cases individuals who developed ESKD during 3–15 years of follow-up; therefore, they must have fast kidney function decline (2). Out of 455 proteins examined, elevated plasma concentrations at baseline of 46 proteins were strong predictors of ESKD risk. In pathway analysis, 20 of these proteins enriched apoptosis processes and TNF receptor signaling pathways as the major disease processes underlying early fast development of ESKD.

To explore the mechanisms that might be involved in this disease process, we reanalyzed snRNA-Seq data of kidney biopsies from healthy individuals and those with DKD (19–21). We specifically focused on healthy and injured (PT_VCAM1-positive) proximal tubular cells. Significant overexpression of genes encoding apoptosis/TNF receptor proteins was observed in the PT_VCAM1-positive cells. Furthermore, overexpression of these genes was positively correlated with an overall index of expression of 205 apoptotic genes in PT_VCAM1-positive cells. Taken together, these findings allow us to hypothesize that individuals with diabetes at high risk of early fast progression to ESKD have an increased proportion of injured proximal tubule cells that are more likely to die triggering “escape” of the apoptosis/TNF receptor proteins into the circulation, thereby increasing the concentration of these proteins in the circulation (Figure 6E). In keeping with this hypothesis, a recent mouse model of acute kidney injury found that, although the PT_VCAM1-positive cells may retain the ability to repair themselves, many of them experienced cell death (22). Other injured kidney cell types also characterize DKD, and these cells could also encode apoptosis/TNF receptor proteins. Notably, the Kidney Precision Medicine Project (KPMP) (23) previously described a subpopulation of maladaptive epithelial cells in the thick ascending limb that share characteristics with injured proximal tubule cells.

In contrast to the above results, using direct sequencing of mRNA obtained from peripheral WBC collected at baseline, 11 of the 46 genes (including 2 apoptosis/TNF receptor genes) encoding the proteins associated with ESKD risk were not expressed in WBC and none of the remaining genes showed different expression in individuals who subsequently did and did not develop ESKD. These negative findings contrasted with significant statistical differences in concentration of some of these proteins in plasma obtained at the same time as WBC, leading us to conclude that the high concentrations of ESKD risk–associated proteins in circulation cannot be accounted for by increased transcription of these genes in WBC. However, considering that secretion of proteins into circulation can be regulated by mechanisms other than transcription, we cannot exclude the possibility that WBC can be a source of increased concentrations in plasma for some of the proteins.

Our study design did not allow us to investigate other organs/tissues/cells as the source of the increased concentrations in circulation of ESKD risk–associated proteins. Presently, there is no literature on this topic to our knowledge, and we found no publications that would inform us why the majority are receptors among circulating ESKD risk–associated proteins and few are ligands or enzymes. However, we provided indirect evidence that the 20 apoptosis/TNF receptor proteins (all receptors) are more important in progression to ESKD than the other 26 ESKD risk–associated proteins. This evidence came from our analysis regarding the usefulness of these proteins to predict ESKD risk. Using an unbiased statistical approach, we identified a prediction model of ESKD risk that included 5 of the 20 apoptosis/TNF receptor proteins (apoptosis score) that was as efficient as the model that included all 46 ESKD risk–associated proteins. Overall, adding the apoptosis score to baseline eGFR, urinary ACR, and HbA_1c_ significantly improved prediction of ESKD risk. This finding has 2 implications. First, the increased concentrations of apoptosis/TNF receptor proteins in circulation can be interpreted as evidence that apoptosis processes and TNF receptor signaling pathways initiate/underlie the fast development of ESKD. Second, apoptosis score can be used in the clinical setting to identify individuals at high risk of the fast development to ESKD and to monitor the intensity of disease processes that underly this development. We developed several equally efficient alternative apoptosis scores using a combination of 5–6 different apoptosis/TNF receptor proteins. This happened because the 20 apoptosis/TNF receptor proteins represented activation of 1 underlying disease processes, and they were intercorrelated.

There are multiple mechanisms of eucaryotic cell death reported in the literature. Historically, 3 different categories of cell death were distinguished: apoptosis, autophagy, and necrosis. A recent nomenclature committee published recommendations that considers not only morphological characteristics but also molecular mechanisms of regulated death cell (24, 25). This resulted in identification of new categories of regulated cell death as well as in distinction of apoptosis due to intrinsic and extrinsic mechanisms and distinction of necrosis to necroptosis, pyroptosis, and ferroptosis. Of importance is that extrinsic apoptosis and necroptosis involve multiple receptors (24), referred to in this study as the apoptosis/TNF receptors, which were found to be the ESKD risk–associated circulating proteins. Unfortunately, the DAVID software had limited abilities to differentiate among the new subcategories of regulated cell death. In 3 recent reviews, the importance of different subcategories of necrosis — i.e., necroptosis, pyroptosis, and ferroptosis — were emphasized as possible mechanisms of the development and progression of kidney diseases (26–28). A few recent publications reviewed the role of regulated cell death in DKD (29, 30). Most of the cited research was derived from cellular or animal studies; there has not been much research done on this topic in humans.

The findings and conclusions of the current study differ from those in one of our previous targeted SOMAscan proteomics studies (7). In that study, which focused on inflammatory proteins (*n* = 194), we identified a set of 17 circulating proteins we referred to as the kidney risk inflammatory score (KRIS) that were strongly associated with ESKD risk. Six of these circulating proteins were TNF receptors. We demonstrated that the elevated concentrations of these KRIS proteins in the circulation were not related to retention due to impaired kidney function. In addition, analysis of bulk RNA-Seq data from dissected tubules and glomeruli from kidney biopsies of individuals who had different stages of DKD revealed no statistically significant differences in expression of genes encoding the KRIS proteins. These findings led us to postulate that elevated levels of KRIS proteins in circulation were derived from overproduction of these proteins in organs other than the kidneys. In the present study, we used more advanced snRNA-Seq to profile gene expression in specific kidney cell types, and we found significant overexpression of genes encoding apoptosis/TNF receptor proteins in injured versus healthy proximal tubule cells. Notably, when we analyzed all proteins measured by SOMAscan platform in our previous studies (10), we found 40 circulating proteins (including the KRIS proteins) that were associated with ESKD risk but did not enrich inflammatory pathways and, instead, only enriched the apoptosis/TNF receptor signaling pathways.

Previously, by comprehensively studying the association between known circulating TNF superfamily ligands and receptors (25 proteins) and the development of early progressive kidney decline in T1D, we observed profiles of 16 circulating TNF receptors associated with early progressive kidney decline that resembled profiles observed in autoimmune disorders (16). This study, which expands the previous study into T2D and measured 430 additional circulating proteins, confirmed the previous fundings but added 30 more circulating proteins strongly associated with the development of ESKD. The analogy with autoimmune disorders, however, was not confirmed in pathway analysis when all 46 proteins were included. This hypothesis, although intriguing, needs to be investigated in follow-up studies of individuals with autoimmune disorders.

In the interpretation of the findings of this study, strengths of the study design should be emphasized. First, the prospective study design and the size of the study groups were appropriate for a comprehensive assessment of associations between multiple circulating proteins and risk of fast progression to ESKD using OLINK platform. Second, it is important to note that the findings are derived from follow-up observations of individuals with T1D and T2D who had normal or mildly impaired kidney function at baseline and developed ESKD during 3–15 years, representing so-called fast decliners (2). Third, the OLINK assays for quantification of circulating proteins were specific and precise. Specifically, we obtained more precise measurements of several TNF receptors as well as other proteins in comparison with SOMAscan measurements.

The following limitations should also be noted. The generalization of our findings is uncertain to individuals with diabetes who are at risk of the development of ESKD as a result of slow kidney function decline, which required 15–40 years of follow-up (2). Our etiological “escape” hypothesis needs to be examined further in animal studies and clinical trials. Also, the prognostic models that included “apoptosis score” should be validated in other cohorts.

Finally, there are several questions that this study did not answer. First, we did not establish the role of the 26 other circulating proteins associated with ESKD risk. This study measured concentrations of circulating proteins only at baseline. Therefore, it is not known whether elevated concentrations of these proteins were in existence from the onset of diabetes or whether they were induced subsequently and in what order; the apoptosis/TNF receptor proteins first and the other proteins later. Answering this question could help to establish which proteins were the possible initiators and which ones the consequences of the disease process that underlie the development of ESKD. Indeed, we recently reported that short-term changes in concentrations of circulating proteins, including those associated with activation of apoptotic processes/TNF receptor signaling pathways, enhanced prediction of fast development of ESKD. Second, in the current OLINK study as well as in the previous SOMAscan study, we found elevated levels of apoptosis/TNF receptor proteins predicting ESKD risk; however, there was lack of elevated concentrations of ligands (chemokines/cytokines) for these receptors as predictors of ESKD risk. It is unclear whether this negative finding has biological significance or if it is a result of a very low concentration of certain ligands in circulation and OLINK as well as SOMAscan assays were unable to measure them. Third, we did not establish whether there are additional sources of elevated concentrations in circulation of the ESKD risk–associated proteins. This study examined only 2 possible hypotheses. Other organ tissues should be examined particularly to establish the sources for the elevations of the other 26 proteins. Fourth, although many proteins associated with the development of ESKD in individuals with DKD found in our studies overlapped with circulating proteins associated with progressive kidney function decline in general populations, it is not clear whether the same pathways are enriched in kidney disease due to different causes. It is of interest to point out that circulating proteins found to be associated with sickle cell–related kidney disease (Figure 2B) and measured by OLINK platform did not enrich the apoptosis/TNF-receptor signaling pathways found in our study (18).

In conclusion, there are many circulating proteins that predict risk of the development of ESKD in diabetes. The number of these proteins will continue to increase as the number of proteins examined on high-throughput proteomics platforms increases. However, many of these proteins are highly intercorrelated and represent the same underlying disease processes or their consequences. Using the functional annotation clustering in DAVID software, we identified apoptosis processes and TNF receptor signaling pathways as possible disease processes related to fast development of ESKD. Further research is needed to validate our findings. This should lead to the development of new therapies that will target specific disease-related pathways and the discovery of effective biomarkers to screen for individuals at risk of the development of ESKD.

## Methods

### Sex as a biological variable.

Our study examined males and females; similar findings are reported for both sexes.

### Study design.

Participants for this research were selected from among 3,500 individuals who were enrolled into the JKS between 1991 and 2009 and were followed until 2016. Instead of studying whole cohorts, we conducted 2 nested case-control studies of about 200 individuals each. This approach significantly reduced the number of individuals for whom costly proteomics measurements were performed. Description of the JKS, selection of individuals, and detailed protocols used are provided in Figure 1 and in Supplemental Methods.

### Selection of individuals for current nested case-control studies.

The current research consists of 2 case-control studies nested in the JKS cohort; one included individuals with T1D and Macro-albuminuria (T1D study exploratory panel) and the other included individuals with T2D and Albuminuria (Macro and Micro) (T2D study validation panel) (Figure 1). Individuals for the T1D nested case-control study were selected from among 522 T1D individuals with Macro-albuminuria enrolled into the JKS. We selected as cases individuals (*n* = 103) who had baseline eGFR ≥ 45 mL/min/1.73 m^2^ and progressed to ESKD during 7–15 years of follow-up. As controls for these cases, we randomly selected 93 individuals from those who had Macro-albuminuria and eGFR ≥ 45 mL/min/1.73 m^2^ at baseline but did not progress to ESKD during the follow-up. This nested case-control study was used previously to examine the association of 25 specific TNF receptor–related proteins with risk of ESKD (16). Individuals for the T2D nested case-control study were selected from among 658 individuals with albuminuria who participated in the JKS. From among those individuals, we selected those (*n* = 40) who had baseline eGFR ≥ 45 mL/min/1.73 m^2^ and progressed to ESKD during 7–15 years of the follow-up as cases. As controls, we randomly selected 163 individuals from those with baseline Albuminuria and eGFR ≥ 45 mL/min/1.73 m^2^ who did not progress to ESKD during the follow-up. All relevant clinical and research data as well as baseline plasma specimens archived in –85°C for individuals selected for the studies, previously collected during the Joslin Kidney Study, were available for this research.

### Proteomics platform.

The OLINK Proseek Multiplex panels were used to measure proteins in archived plasma samples using qPCR through PEA technology (6). In total, we measured 455 proteins contained on 5 OLINK panels: Cardiovascular II, Cardiovascular III, Development, Neurology, and Oncology II. The measurements were performed at the OLINK laboratory and were presented as relative values (Normalized Protein Expression [NPX]) on a log_2_-scale. Quality control (QC) was performed in 2 steps: (a) each sample plate was evaluated on the SD of the internal controls, and (b) the quality of each sample was assessed by evaluating the deviation from median value of the internal controls. Proportions of samples passing QC were 92%–100% and 96%–100% in T1D and T2D studies, respectively. Average intraassay percentages of coefficients of variation in T1D and T2D studies were 4%–21% and 11%–18%, respectively. 

### Pathway analyses.

For pathway enrichment analysis of the differentially expressed proteins, we applied the DAVID 6.8. The significant proteins were submitted to the software, and all the measured proteins annotated in the databases were considered as the background proteins. Enriched pathways in the DAVID databases (e.g., GO, KEGG, REACTOME pathways and UniprotKB Keywords) were clustered into functional categories using DAVID functional annotation clustering (31). The enrichment score of each clustered group was calculated by the geometric mean (in –log scale) of the Expression Analysis Systemic Explorer (EASE) scores (modified Fisher exact *P* values) (32) associated with the enriched annotation terms that belong to the gene group (31). The enrichment scores were used to rank their biological significance, and a higher score for a group indicates that the group members are enriched. For each cluster, pathways with EASE scores of *P* < 0.01 are shown in the figure as significantly enriched database-specific pathways in the databases.

### Sequencing of mRNA from peripheral WBC.

Peripheral blood was collected from a subset of individuals enrolled in the JKS (23 T1D and 48 T2D) using PAXgene Blood RNA tubes (2.5 mL whole blood) (PreAnalytiX) and were stored at –85°C until analysis. Total RNA was isolated using the PAXgene RNA kit according to the manufacturer’s protocol. The quality and integrity of the isolated RNA was evaluated using the 2100 Bioanalyzer platform with Agilent RNA 6000 Nano Kit (Agilent Technologies). RNA samples were submitted to the High-Throughput Genomics Shared Resource at the Huntsman Cancer Institute at the University of Utah for library preparation and sequencing. RNA-Seq libraries were prepared using the Illumina Stranded Total RNA Library Prep with Ribo-Zero Plus Kit. Pooled libraries were sequenced across an S4 flow cell on a NovaSeq 6000 (Illumina) using the NovaSeq S4 Reagent Kit v1.5, targeting a median sequencing depth of 2,500 million 150 bp paired-reads per lane, and demultiplexed FASTQ files were generated. Sequencing reads were then aligned to the GRCh38/hg38 reference genome using STAR-2.7.6a (33). Read pairs mapping to genes annotated using Ensembl release 102 were counted using featureCounts v1.6.3 (34). Differentially expressed genes (DEGs) between individuals who did and did not progress to ESKD during follow-up were determined using the R software packages, hciR and DESeq2 (35). A Bonferroni-adjusted *P* < 0.05 was used as the significance threshold to determine DEGs.

### Single-cell sequencing of kidney cells.

snATAC-Seq quantifies chromatin accessibility in individual cells. These technologies can interrogate transcriptional and chromatin accessibility states and signaling pathways in multiple cell types in health and disease. We downloaded a previously published single-cell atlas of DKD to compare cell-specific DEGs and differentially accessible chromatin regions (DAR) for genes encoding our circulating ESKD risk–associated proteins (19–21). The snRNA-Seq and snATAC-seq libraries were generated from kidney cortex samples obtained from 6 control adults and 7 with DKD. These individuals ranged in age from 50 to 78 years (median 57 years) and included 7 men and 6 women. Mean eGFR of DKD donors (66 ± 25 mL/min/1.73 m^2^) and control samples (74 ± 15 mL/min/1.73 m^2^) was not statistically different. Two donors with DKD had mild to moderate proteinuria and moderate interstitial fibrosis and glomerulosclerosis identified on histology. Cell-specific DEG and DAR for VCAM1^+^ proximal tubule cells (PT_VCAM1) compared with control proximal tubule were downloaded from ref. 19 and intersected with our list of 46 circulating ESKD risk–associated proteins or a list of hallmark apoptosis genes (*n* = 205) obtained from the msigdbr R package (v7.5.1) (20). For the correlation between genes encoding the 46 circulating proteins and apoptosis genes, we downloaded the aggregated snRNA-Seq seurat object from a publicly available website on cellxgene (https://cellxgene.cziscience.com/collections/b3e2c6e3-9b05-4da9-8f42-da38a664b45b). The snRNA-Seq RNA assay was log-normalized using the NormalizeData function in Seurat (v4.1.0). Hallmark apoptosis was summed for each PT_VCAM1 cell and divided by the total number of transcripts per cell to create an apoptosis index. We computed a Pearson correlation between the apoptosis index and transcript counts for genes encoding the 46 ESKD risk–associated proteins using the rcorr function in the Hmisc R package (v4.7.0) and visualized results using ggplot2. We imputed missing expression data using Markov Affinity-based Graph Imputation of Cells (MAGIC) (36) and performed the same correlation analysis with the imputed data. Subsequently, we used a linear mixed effect model to quantify the effect size for all protein-coding genes in a linear regression model using apoptosis index as the dependent variable. A mixed effect for each sample was used to control for nonindependence of single-cell measurements within each donor using the lmer package in R (37). We computed a density estimate of the gene effect sizes to compare all protein-coding genes to the subset of 46 circulating proteins.

### Statistics.

Baseline characteristics were presented as median and interquartile range or number and percent, as applicable. To correct for multiple testing, Bonferroni-adjusted *P* values were calculated from 455 proteins in T1D and from the selected proteins in T2D, respectively. Bonferroni-adjusted *P* < 0.01 was considered statistically significant for investigating proteins in T1D and T2D. To consider the intervariation between batches, we applied batch-specific quartiles to proteins in each batch. Univariate and multivariable logistic regression models were used to estimate the effect of proteins on the onset of ESKD within 15 years. Multivariable models were adjusted for baseline eGFR, urinary ACR, HbA_1c_, and study indicator (T1D or T2D) in the combined studies of T1D and T2D. Heterogeneity of the effects of proteins on onset of ESKD between the studies were assessed by random effects model using I², after adjusting for eGFR, ACR, and HbA_1c_ in each study.

We examined to what degree the proteins could improve prediction of onset of ESKD within 15 years. We applied LASSO logistic regression for protein selection by batch-specific quartiles using glmnet R package. This method penalized the sum of the absolute values of the regression coefficients, leading to some coefficients shrinking to zero and thus simultaneously performed variable selection (38). A penalty factor for penalized maximum likelihood estimation was chosen by 10-fold cross-validation at its minimum level. We developed an apoptosis score with the selected proteins through LASSO, using a logistic regression model of onset of ESKD within 15 years. The apoptosis score was computed as a sum of levels of the contributing proteins with batch-specific quartiles weighted by their β coefficients, according to the following formula:

Apoptosis score = β_A_ × A + β_B_ × B + β_C_ × C + …

Model performance was evaluated in risk discrimination with c statistic. In addition, 2 alternative measures of risk discrimination were calculated: one is the category-free net reclassification index (NRI) (39), and the other is the relative integrated discrimination improvement (rIDI) (40). The analyses testing the improvement in predictive performance were computed using logistic regression models with methods developed by Kennedy and Pencina (40).

To examine possible determinants of variation of the apoptosis score, we estimated the OR for onset of ESKD within 15 years according to the apoptosis score, regarded as a categorical variable, by its quartiles without and with adjustment for relevant covariates pattern 1 (eGFR, ACR, and HbA_1c_) and 2 (1 + Age and Systolic blood pressure) among all individuals in the combined studies. Cumulative incidence of ESKD according to the quartiles of apoptosis score was examined using time-to-event analysis. Two-sided *P* < 0.05 was considered as statistical significance through the analyses. All analyses were performed by SAS 9.4 (SAS Institute Inc.), Stata 15 (StataCorp.), and R version 4.0.3 (R Core Team [2020]).

### Study approval.

The Joslin Diabetes Center Committee on Human Studies approved the informed consent, recruitment, and examination procedures for the JKS.

### Data availability.

The datasets that support the findings of this study are available in a deidentified manner for collaborative research from the corresponding author upon request. The RNA-Seq data discussed in this publication have been deposited in National Center for Biotechnology Information’s (NCBI’s) Gene Expression Omnibus (GEO) and are accessible through GEO series accession no. GSE271708 (https://www.ncbi.nlm.nih.gov/geo/query/acc.cgi?acc=GSE271708). The code for statistical analysis of single-cell data is publicly available in GitHub at the following link: https://github.com/p4rkerw/Ihara_JCI_Insight_2024 (ID: 7cf969af956ca3dbaca3b35e92f205d34c0f55f7).

## Author contributions

KI contributed to design of the study, contributed to the proteomic data collection in the JKS, performed data analysis, and wrote the first revision of the manuscript. BK, ES, HK, ZIMD, and JR, contributed to collection of data in the JKS, implementation of experiments/measurements, and analysis of data and reviewed the manuscript. PCW and BDH analyzed and interpreted the results of snRNA-Seq and contributed to the editing of manuscript. JW and KD, contributed to interpretation of the results and to the editing of the manuscript. JMD contributed to interpretation of the results of pathway analysis and reviewed the manuscript. MAN, AD, and RGN contributed to interpretation of the results and to the editing of the manuscript. MGP is responsible for sequencing of RNA of peripheral WBC, interpretation of the results, and editing of the manuscript. ASK designed and supervised the study implementation, planned and contributed to the data analysis, contributed to interpretation of the results, and contributed to the manuscript writing.

## Supplementary Material

Supplemental data

Supporting data values

## Figures and Tables

**Table 2:**
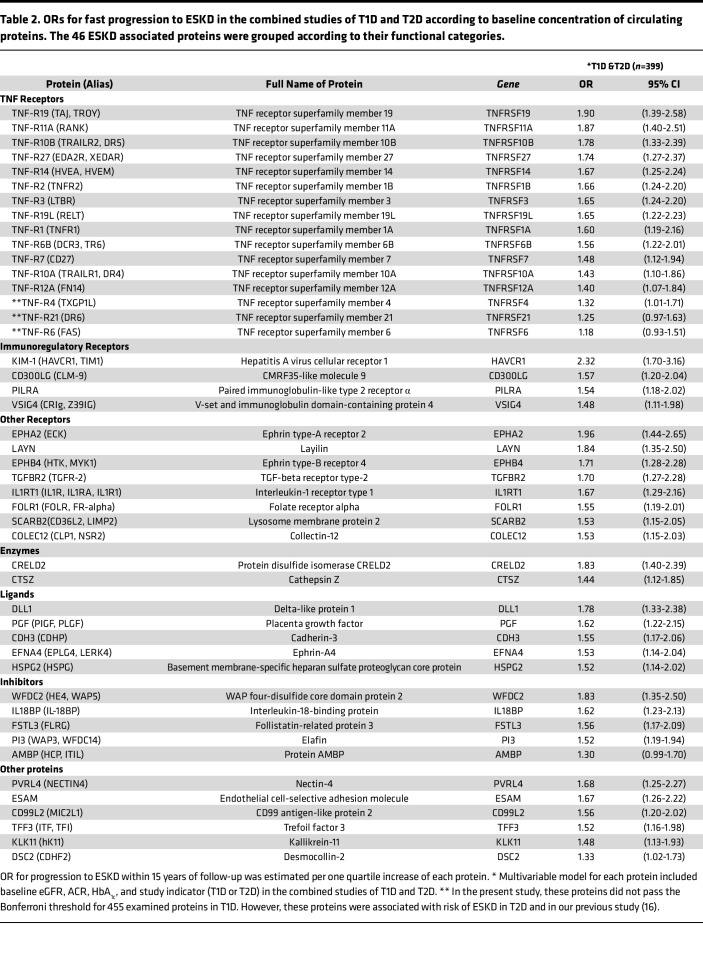
ORs for fast progression to ESKD in the combined studies of T1D and T2D according to baseline concentration of circulating proteins. The 46 ESKD associated proteins were grouped according to their functional categories.

**Table 1:**
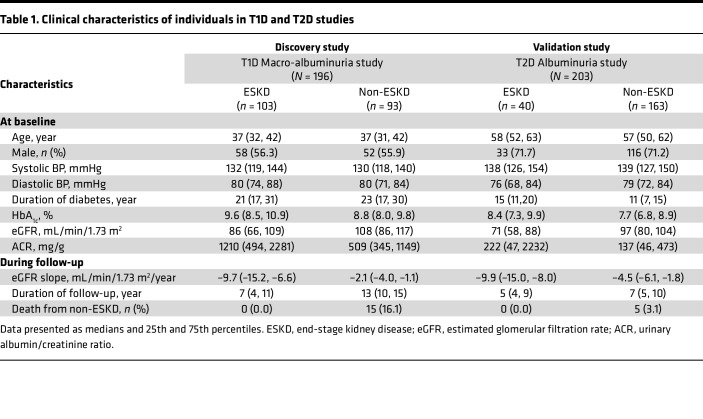
Clinical characteristics of individuals in T1D and T2D studies

**Table 3:**
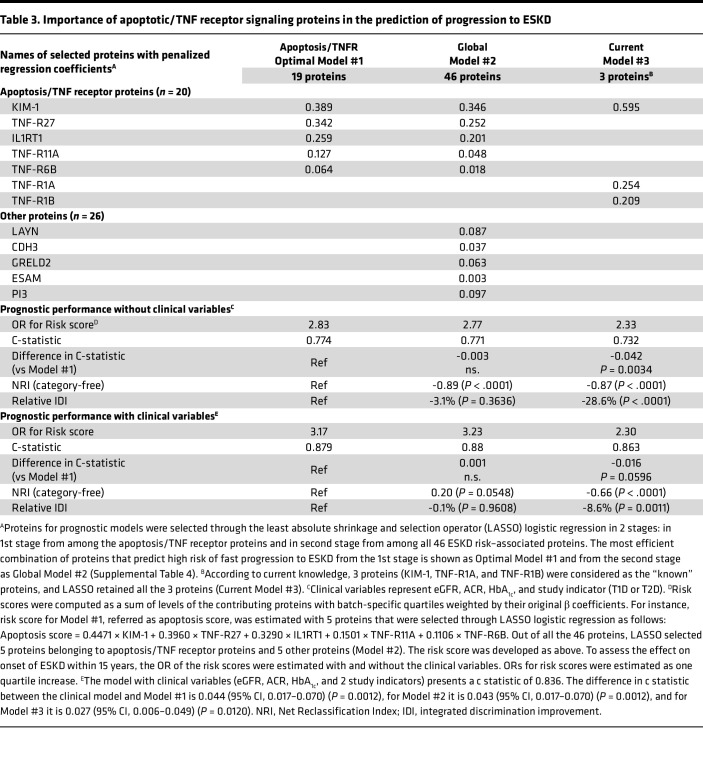
Importance of apoptotic/TNF receptor signaling proteins in the prediction of progression to ESKD

## Version 1 10/22/2024

Electronic publication
