# Supplementary material for: Circulating proteins linked to apoptosis processes and fast development of end-stage kidney disease in diabetes
Source: JCI Insight. 2024 Oct 22;9(20):e178373. doi: 10.1172/jci.insight.178373 (PMC11529980; doi:10.1172/jci.insight.178373)
Supplement: Supplemental data [file jciinsight-9-178373-s266.pdf]

Table of Contents for the Supplementary Material

Appendix for methods

Table S1. ORs for fast progression to ESKD in T1D and T2D studies according to baseline concentration of circulating proteins. The 46 ESKD associated proteins were grouped according to their functional categories.

Table S2. Results of Pathway analysis for the 46 ESKD risk associated proteins measured by OLINK proteomics platform.

Table S3. Characteristics of study groups used for examination of mRNA expression in white blood cells of genes encoding 46 ESKD associated circulating proteins.

Table S4. Selection* of prognostic models and their performances in predicting development of ESKD.

Figure S1. All protein coding genes were and their effect size on apoptosis index.

Figure S2. Differential chromatin accessibility of genes encoding for 46 circulating ESKD risk associated proteins in PT_VCAM1 vs. control proximal tubule cells.

Figure S3. Pathway analysis for SOMAscan results. Reanalyzed data from our previous publication (S5).

Appendix for methods:

Participants for this research were selected from among individuals who were enrolled in the Joslin Kidney Study (See **Figure 1**). Instead of studying whole cohorts, we conducted two nested case-control studies of about 200 individuals each. This approach significantly reduced the number of individuals for whom costly proteomics measurements were performed.

Joslin Kidney Study (JKS)

The Joslin Clinic is a large 120-year-old referral center for treatment of individuals with diabetes. These individuals are mainly residents of Eastern Massachusetts and are referred to the Clinic because of type 1 diabetes (T1D) or type 2 diabetes (T2D) diagnoses. The majority are referred in early stages of diabetes and remain under the care of the clinic for a long time. The JKS is a longitudinal observational study that aims to investigate the determinants of, and

to describe the natural history of kidney function decline in, T1D and T2D in the Joslin Clinic population. The Joslin Diabetes Center Committee on Human Studies approved the informed consent, recruitment, and examination procedures for the JKS.

The JKS aimed to enroll all eligible participants with albuminuria and a similar number of individuals taken randomly from a much larger pool of eligible individuals with normoalbuminuria. The median values of albumin-to-creatinine ratio (ACR) from 2 or more consecutive urine samples obtained during the 2-year period preceding enrollment into the JKS were used to grouped participants into: Macro-Albuminuria (ACR ≥300 mg/g), Micro-Albuminuria (30≤ ACR <300 mg/g), and Normo-Albuminuria (ACR <30 mg/g) for purpose of selecting individuals into the JKS.

Briefly, 1,884 individuals with T1D were enrolled from among 3,500 adults aged 20-64 years who attended the Joslin Clinic between 1991 and 2009. Similarly, 1,474 individuals with T2D were recruited from among 9,000 adults aged 35-64 years who attended the Joslin Clinic between 2003 and 2009. All enrolled individuals were followed for up to 15 years unless they died due to unrelated-ESKD deaths or were lost to follow-up. All enrolled individuals had baseline and biannual follow-up examinations with blood and urine specimens taken for laboratory determinations and BioBank storage in -85°C. Individuals with less frequent clinic visits and those who stopped coming to the clinic were examined at their homes. The goal of follow-up was to ascertain eGFR slope and date of onset of ESKD or death.

Measurements of serum creatinine performed at routine clinic visits or during special examinations were used to determine kidney function at baseline and its changes during follow-up. Protocols to calibrate serum creatinine measurements over time were described previously (S1). The Chronic Kidney Disease Epidemiology Collaboration formula was used to estimate eGFR (S2). Description of other measurements of clinical characteristics performed in the JKS were included in previous publications (S1, S3, S4).

To identify ESKD and deaths in individuals enrolled into the JKS, we queried the United States Renal Data System (USRDS) and the National Death Index (NDI) covering all events up to the end of 2018. The USRDS maintains a roster of US patients receiving renal replacement therapy that includes dates of dialysis and transplantation. The NDI is a comprehensive roster of deaths in the United States that includes the date and cause of death.

Selection of individuals for nested case-control studies (see Figure 1):

The current research consists of two case-control studies nested in the JKS cohort, one included individuals with T1D and Macro-Albuminuria (T1D study exploratory panel) and the other included individuals with T2D and Albuminuria (Macro and Micro) (T2D study validation panel). Individuals for the T1D nested case-control study were selected from among 522 T1D individuals with Macro-albuminuria enrolled into the JKS. There were 103 individuals (cases) available for the study who had baseline eGFR >45 ml/min/1.73m^2 and developed ESKD during 15 years of follow-up as cases. As controls for these cases, we randomly selected 93 individuals from those who had Macro-albuminuria and eGFR >45 ml/min/1.73m^2 at baseline but did not progress to ESKD during follow-up. The nested case-control study of individuals with T1D was used previously to examine the association of 25 tumor necrosis factor (TNF) receptor related proteins with risk of ESKD (S1). Individuals for the T2D nested case-control study were selected from among 658 individuals with albuminuria who participated in the JKS. From among those individuals, there were 40 individuals available as cases for the study who had had baseline eGFR >45 ml/min/1.73m^2 and progressed to ESKD during 15 years of follow-up. As controls, we randomly selected 163 individuals from those with baseline albuminuria and eGFR >45 ml/min who did not progress to ESKD during follow-up. All relevant clinical and research data as well as baseline plasma specimens archived in -85°C for individuals selected for the studies were available for this research.

Supplementary references

S1. Ihara K, et al. A profile of multiple circulating tumor necrosis factor receptors associated with early progressive kidney decline in Type 1 Diabetes is similar to profiles in autoimmune disorders. *Kidney Int*. 2021;99(3):725-736.

S2. Levey AS, et al. A new equation to estimate glomerular filtration rate. *Ann Intern Med.* 2009;150(9):604-612.

S3. Niewczas MA, et al. A signature of circulating inflammatory proteins and development of end-stage renal disease in diabetes. *Nat Med.* 2019;25(5):805-813.

S4. Satake E, et al. Comprehensive Search for Novel Circulating miRNAs and Axon Guidance Pathway Proteins Associated with Risk of ESKD in Diabetes. *J Am Soc Nephrol.* 2021;32(9):2331-2351.

S5. Kobayashi H, et al. Results of untargeted analysis using the SOMAscan proteomics platform indicate novel associations of circulating proteins with risk of progression to kidney failure in diabetes. *Kidney Int.* 2022;102(2):370-381.

Table S1. ORs for fast progression to ESKD in T1D and T2D studies according to baseline concentration of circulating proteins. The 46 ESKD associated proteins were grouped according to their functional categories.

			Univariate T1D study (n=196)		Univariate T2D study (n=203)		Multivariable analysis** Combined T1D &T2D (n=399)	
**Protein (Alias)**	**Full Name of Protein**	**Gene**	OR	P $_{Bonferroni}$	OR	P $_{Bonferroni}$	OR	95% CI
**25 Tumor Necrosis Factor (TNF) Receptors were measured**								
**TNF-R19 (TAJ, TROY)**	**TNF receptor superfamily member 19**	***TNFRSF19***	2.06	0.0005	5.28	<.0001	1.90	(1.39-2.58)
**TNF-R11A (RANK)**	**TNF receptor superfamily member 11A**	***TNFRSF11A***	2.15	0.0002	3.71	<.0001	1.87	(1.40-2.51)
**TNF-R10B (TRAILR2, DR5)**	**TNF receptor superfamily member 10B**	***TNFRSF10B***	2.49	<.0001	2.75	0.0002	1.78	(1.33-2.39)
**TNF-R27 (EDA2R, XEDAR)**	**TNF receptor superfamily member 27**	***TNFRSF27***	2.12	0.0003	3.68	<.0001	1.74	(1.27-2.37)
**TNF-R14 (HVEA, HVEM)**	**TNF receptor superfamily member 14**	***TNFRSF14***	2.02	0.0006	3.34	<.0001	1.67	(1.25-2.24)
**TNF-R2 (TNFR2)**	**TNF receptor superfamily member 1B**	***TNFRSF1B***	1.82	0.0098	4.01	<.0001	1.66	(1.24-2.20)
**TNF-R3 (LTBR)**	**TNF receptor superfamily member 3**	***TNFRSF3***	2.02	0.0006	3.18	<.0001	1.65	(1.24-2.20)
**TNF-R19L (RELT)**	**TNF receptor superfamily member 19L**	***TNFRSF19L***	2.06	0.0004	2.85	<.0001	1.65	(1.22-2.23)
**TNF-R1 (TNFR1)**	**TNF receptor superfamily member 1A**	***TNFRSF1A***	1.86	0.0058	4.07	<.0001	1.60	(1.19-2.16)
**TNF-R6B (DCR3, TR6)**	**TNF receptor superfamily member 6B**	***TNFRSF6B***	1.97	0.0017	2.10	0.0028	1.56	(1.22-2.01)
**TNF-R7 (CD27)**	**TNF receptor superfamily member 7**	***TNFRSF7***	1.97	0.0017	2.57	0.0002	1.48	(1.12-1.94)
**TNF-R10A (TRAILR1, DR4)**	**TNF receptor superfamily member 10A**	***TNFRSF10A***	1.85	0.0085	2.38	0.0006	1.43	(1.10-1.86)
**TNF-R12A (FN14)**	**TNF receptor superfamily member 12A**	***TNFRSF12A***	1.86	0.0086	2.33	0.0004	1.40	(1.07-1.84)
***TNF-R4 (TXGP1L)**	**TNF receptor superfamily member 4**	***TNFRSF4***	1.64	0.1712	2.49	0.0002	1.32	(1.01-1.71)
***TNF-R21 (DR6)**	**TNF receptor superfamily member 21**	***TNFRSF21***	1.49	1.0000	2.79	<.0001	1.25	(0.97-1.63)
***TNF-R6 (FAS)**	**TNF receptor superfamily member 6**	***TNFRSF6***	1.43	1.0000	1.92	0.0110	1.18	(0.93-1.51)
**47 Immunoregulatory Receptors were measured**								
**KIM1 (HAVCR1, TIM1)**	**Hepatitis A virus cellular receptor 1**	***HAVCR1***	2.84	<.0001	3.88	<.0001	2.32	(1.70-3.16)
**CD300LG (CLM-9)**	**CMRF35-like molecule 9**	***CD300LG***	1.86	0.0058	3.07	<.0001	1.57	(1.20-2.04)
**PILRA**	**Paired immunoglobulin-like type 2 receptor α**	***PILRA***	1.97	0.0011	2.38	0.0006	1.54	(1.18-2.02)
**VSIG4 (CRIg, Z39IG)**	**V-set and immunoglobulin domain-containing protein 4**	***VSIG4***	1.97	0.0011	2.60	0.0003	1.48	(1.11-1.98)
**71 Other Receptors were measured**								
**EPHA2 (ECK)**	**Ephrin type-A receptor 2**	***EPHA2***	2.15	0.0002	4.15	<.0001	1.96	(1.44-2.65)
**LAYN**	**Layilin**	***LAYN***	2.17	0.0002	3.87	<.0001	1.84	(1.35-2.50)
**EPHB4 (HTK, MYK1)**	**Ephrin type-B receptor 4**	***EPHB4***	1.82	0.0098	5.47	<.0001	1.71	(1.28-2.28)

**TGFBR2 (TGFR-2)**	**TGF-beta receptor type-2**	***TGFBR2***	2.06	0.0005	3.55	<.0001	1.70	(1.27-2.28)
**IL1RT1 (IL1R, IL1RA, IL1R1)**	**Interleukin-1 receptor type 1**	***IL1RT1***	2.02	0.0006	2.90	<.0001	1.67	(1.29-2.16)
**FOLR1 (FOLR, FR-alpha)**	**Folate receptor alpha**	***FOLR1***	1.93	0.0029	2.66	<.0001	1.55	(1.19-2.01)
**SCARB2(CD36L2, LIMP2)**	**Lysosome membrane protein 2**	***SCARB2***	1.86	0.0086	3.31	<.0001	1.53	(1.15-2.05)
**COLEC12 (CLP1, NSR2)**	**Collectin-12**	***COLEC12***	2.06	0.0004	2.47	0.0002	1.53	(1.15-2.03)
**71 Enzymes were measured**								
**CRELD2**	**Protein disulfide isomerase CRELD2**	***CRELD2***	2.30	<.0001	2.44	0.0007	1.83	(1.40-2.39)
**CTSZ**	**Cathepsin Z**	***CTSZ***	1.97	0.0011	2.11	0.0020	1.44	(1.12-1.85)
**110 Ligands were measured**								
**DLL1**	**Delta-like protein 1**	***DLL1***	2.26	0.0001	3.37	<.0001	1.78	(1.33-2.38)
**PGF (PIGF, PLGF)**	**Placenta growth factor**	***PGF***	1.93	0.0029	3.27	<.0001	1.62	(1.22-2.15)
**CDH3 (CDHP)**	**Cadherin-3**	***CDH3***	1.94	0.0029	2.77	<.0001	1.55	(1.17-2.06)
**EFNA4 (EPLG4, LERK4)**	**Ephrin-A4**	***EFNA4***	1.90	0.0051	3.52	<.0001	1.53	(1.14-2.04)
**HSPG2 (HSPG)**	**Basement membrane-specific heparan sulfate proteoglycan core protein**	***HSPG2***	1.86	0.0058	2.54	0.0001	1.52	(1.14-2.02)
**38 Inhibitors were examined**								
**WFDC2 (HE4, WAP5)**	**WAP four-disulfide core domain protein 2**	***WFDC2***	2.15	0.0002	3.94	<.0001	1.83	(1.35-2.50)
**IL18BP (IL-18BP)**	**Interleukin-18-binding protein**	***IL18BP***	2.10	0.0002	2.70	<.0001	1.62	(1.23-2.13)
**FSTL3 (FLRG)**	**Follistatin-related protein 3**	***FSTL3***	1.97	0.0011	3.19	<.0001	1.56	(1.17-2.09)
**PI3 (WAP3, WFDC14)**	**Elafin**	***PI3***	2.16	0.0001	1.97	0.0037	1.52	(1.19-1.94)
**AMBP (HCP, ITIL)**	**Protein AMBP**	***AMBP***	1.93	0.0029	2.34	0.0012	1.30	(0.99-1.70)
**69 Other proteins were examined**								
**PVRL4 (NECTIN4)**	**Nectin-4**	***PVRL4***	1.93	0.0029	4.64	<.0001	1.68	(1.25-2.27)
**ESAM**	**Endothelial cell-selective adhesion molecule**	***ESAM***	1.86	0.0058	4.15	<.0001	1.67	(1.26-2.22)
**CD99L2 (MIC2L1)**	**CD99 antigen-like protein 2**	***CD99L2***	2.02	0.0006	2.07	0.0034	1.56	(1.20-2.02)
**TFF3 (ITF, TFI)**	**Trefoil factor 3**	***TFF3***	2.06	0.0004	2.29	0.0002	1.52	(1.16-1.98)
**KLK11 (hK11)**	**Kallikrein-11**	***KLK11***	1.85	0.0085	2.73	<.0001	1.48	(1.13-1.93)
**DSC2 (CDHF2)**	**Desmocollin-2**	***DSC2***	1.82	0.0098	2.26	0.0008	1.33	(1.02-1.73)

OR for progression to ESKD within 15 years of follow-up was estimated per one quartile increase of each protein.

Heterogeneity of ORs between the studies was assessed by random effects model using I^2. Only OR for EPHB4 (p=0.013) showed evidence of heterogeneity. OR for other proteins did not show evidence of heterogeneity.

* In the present study, these proteins did not pass the Bonferroni threshold for 455 examined proteins in T1D. However, these proteins were associated with risk of ESKD in T2D and in our previous study (S1).

**Multivariable model for each protein included baseline eGFR, urinary ACR, HbA_{1c}, and study indicator (T1D or T2D)

Table S2. Results of Pathway analysis for the 46 ESKD risk associated proteins measured by OLINK proteomics platform.

**Pathways or Terms**	Count	Frequency (%)	Background frequency (%)	Fold Enrichment	P value
**Cluster 1 (Enrichment score*=6.4)**					
IPR001368:TNFR/NGFR cysteine-rich region	14	30.4	3.6	8.5	8.4E-12
SM00208:TNFR	14	30.4	4.7	7.6	2.5E-11
REPEAT:TNFR-Cys 1	14	30.4	3.8	8.0	3.4E-11
REPEAT:TNFR-Cys 2	14	30.4	3.8	8.0	3.4E-11
REPEAT:TNFR-Cys 3	13	28.3	3.4	8.4	9.2E-11
REPEAT:TNFR-Cys	12	26.1	3.1	8.3	9.4E-10
DOMAIN:TNFR-Cys	11	23.9	2.9	8.2	9.2E-9
REPEAT:TNFR-Cys 4	7	15.2	1.8	8.5	1.8E-5
KW-0053~Apoptosis	14	30.4	12.6	3.2	3.3E-5
hsa04060:Cytokine-cytokine receptor interaction	18	39.1	23.2	2.2	4.0E-4
GO:0006915~apoptotic process	11	23.9	7.8	3.2	7.0E-4
**Cluster 2 (Enrichment Score=3.8)**					
KW-0675~Receptor	27	58.7	37.5	2.1	1.3E-6
TOPO_DOM:Extracellular	31	67.4	39.1	1.7	7.8E-5
TOPO_DOM:Cytoplasmic	32	69.6	41.4	1.7	8.2E-5
TRANSMEM:Helical	32	71.7	43.6	1.6	8.3E-5
KW-0812~Transmembrane	35	73.9	47.7	1.6	2.1E-4
KW-1133~Transmembrane helix	29	73.9	47.7	1.6	2.1E-4
GO:0016021~integral component of membrane	33	63.0	39.4	1.6	9.7E-4
KW-0472~Membrane	33	78.3	56.9	1.4	1.8E-3
GO:0005886~plasma membrane	35	76.1	55.5	1.4	3.0E-3
**Cluster 3 (Enrichment Score=2.3)**					
DOMAIN:TNFR-Cys	11	23.9	2.9	8.2	9.2E-9
GO:0005031~tumor necrosis factor-activated receptor activity	8	17.4	1.8	9.7	5.0E-7
GO:0033209~tumor necrosis factor-mediated signaling pathway	8	17.4	2.2	8.2	4.4E-6
**Cluster 4 (Enrichment Score=1.1)**					
DOMAIN:Death	5	10.9	1.6	6.9	2.5E-3
IPR000488:Death domain	5	10.9	1.8	6.1	4.6E-3
IPR011029:Death-like domain	5	10.9	1.8	6.1	4.6E-3
SM00005:DEATH	5	10.9	2.4	5.4	6.9E-3

*Enrichment scores were calculated by the geometric means (in –log scale) of the Expression analysis systemic explorer (EASE) scores (modified Fisher Exact p-values) for each cluster. ESKD: end-stage kidney disease.

Table S3. Characteristics of study groups used for examination of mRNA expression in white blood cells of genes encoding 46 ESKD risk associated circulating proteins.

	**ESKD (n=16)**	**Non-ESKD (n=55)**
At baseline		
Age, year	51 (28, 60)	51 (43, 58)
T1D / T2D	9 / 7	14 / 41
Male, n (%)	8 (50%)	33 (60%)
Duration of diabetes, year	15 (12, 29)	13 (8, 20)
HbA_{1c}, %	8.4 (8.1, 10.5)	8.1 (6.9, 9.4)
eGFR, ml/min/1.73m^2	77 (62, 107)	101 (88, 112)
ACR, mg/g	555 (158, 942)	70 (25, 415)
During follow-up		
eGFR slope, ml/min/1.73m^2/year	-7.5 (-10.1, -6.0)	-2.0 (-7.7, -0.7)

Data presented as median and inter-quartile range.
ESKD: end-stage kidney disease. T1D: type 1 diabetes. T2D: type 2 diabetes. eGFR: estimated glomerular filtration rate. ACR: albumin-to-creatinine ratio.

Table S4. Selection* of prognostic models and their performances in predicting development of ESKD.

Names of Selected	From 20 proteins	From 19 proteins	From 19 proteins	From 19 proteins	From 19 proteins	From 19 proteins	From 19 proteins	From 46 proteins	Known Biomarkers****
**proteins with penalized regression coefficients***	**Model #1**	**Model #2**	**Model #3**	**Model #4**	**Model #5**	**Model #6**	**Model #7**	**Global model**	**KIM1, TNF-R1A, TNF-R1B**
**TNF receptor/apoptosis proteins (n=20)**									
**KIM1**	0.384	0.389	0.393	0.395	0.428	0.387	Ø	0.346	0.595
**TNF-R27**	0.326	0.342	0.325	0.352	0.325	Ø	0.335	0.252	
**IL1RT1**	0.257	0.259	0.260	0.263	Ø	0.261	0.322	0.201	
**TNF-R11A**	0.112	0.127	0.123	Ø	0.112	0.171	0.164	0.048	
**TNF-R6B**	0.055	0.064	Ø	0.070	0.051	0.037	0.109	0.018	
**TNF-R19**	0.047	Ø	0.065	0.091	0.034	0.156	0.116		
**EPHA2**					0.021	0.031	0.005		
**TNF-R3**					0.064	0.019			
**TNF-R19L**					0.018				
**TNF-R10B**						0.013			
**TNF-R12A**						0.050			
**TNF-R1A**									0.209
**TNF-R1B**									0.254
**Other proteins (n=26)**									
**LAYN**								0.087	
**CDH3**								0.037	
**GRELD2**								0.063	
**ESAM**								0.003	
**PI3**								0.097	
**Prognostic performance**									
Without Clinical variables									
OR for score**	2.71	2.83	2.89	2.86	2.73	2.66	2.62	2.77	2.33
C-statistic	0.776	0.775	0.778	0.777	0.768	0.763	0.760	0.771	0.732

With Clinical variables***									
OR for score**	2.92	3.17	3.24	3.32	3.49	2.82	2.72	3.23	2.30
C-statistic	0.873	0.879	0.880	0.884	0.883	0.878	0.869	0.880	0.861

* Proteins for prognostic models were selected through the least absolute shrinkage and selection operator (LASSO) logistic regression in two stages. In the 1st stage, selection was from among 20 or 19 apoptosis/tumor necrosis factor (TNF) receptors proteins (Models #1 through #7.) To explore whether different combinations of the apoptosis/TNF receptor proteins would have similar prognostic performance to predict risk of end-stage kidney disease (ESKD), we ran LASSO logistic regression and removed proteins one by one from among the apoptosis/TNF receptor proteins and the prognostic performance of the models. ⊘ indicates absence of protein for LASSO selection. When performing the multiple LASSO analyses and removing one protein at a time from the list of 20 proteins, three models (grey-highlighted columns) of these proteins (red-framed) had similar high efficiency to predict fast progression to ESKD. In the 2nd stage, proteins were selected from among all 46 ESKD-associated proteins
According to the previous findings, 3 proteins (KIM-1, TNF-R1A and TNF-R1B) were considered as the “Known Biomarkers”, and LASSO retained all of them for the prognostic model.

To estimate the predictive performance of each model, we created and used the apoptosis score with β coefficients of selected proteins in each model with and without clinical covariates.

** Apoptosis scores were computed as a sum of levels of the contributing proteins with batch-specific quartiles weighted by their original β coefficients. For instance, the apoptosis score for Model #1, was estimated with 5 proteins that were selected through LASSO logistic regression as follows:

Apoptosis score = 0.4471×KIM1 + 0.3960×TNF-R27 + 0.3290×IL1RT1 + 0.1501×TNF-R11A + 0.1106×TNF-R6B.

Out of all 46 proteins, LASSO selected 5 proteins belonging to apoptosis/TNF receptor proteins and 5 other proteins (Model #2). The apoptosis score was developed in similar way as above.
** Clinical variables represent eGFR, ACR, HbA_{1c}, and study indicator (T1D or T2D)

To assess the effect on onset of ESKD within 15 years, OR of the risk score was estimated with and without the clinical variables. ORs of apoptosis score and ACR were estimated as one quartile increase. ORs of eGFR and HbA_{1c} were estimated as 1 ml/min/1.73m^2 and 1% increase, respectively.

LASSO penalizes the sum of the absolute values of regression coefficients, and a predictor with a coefficient of zero was excluded from the model

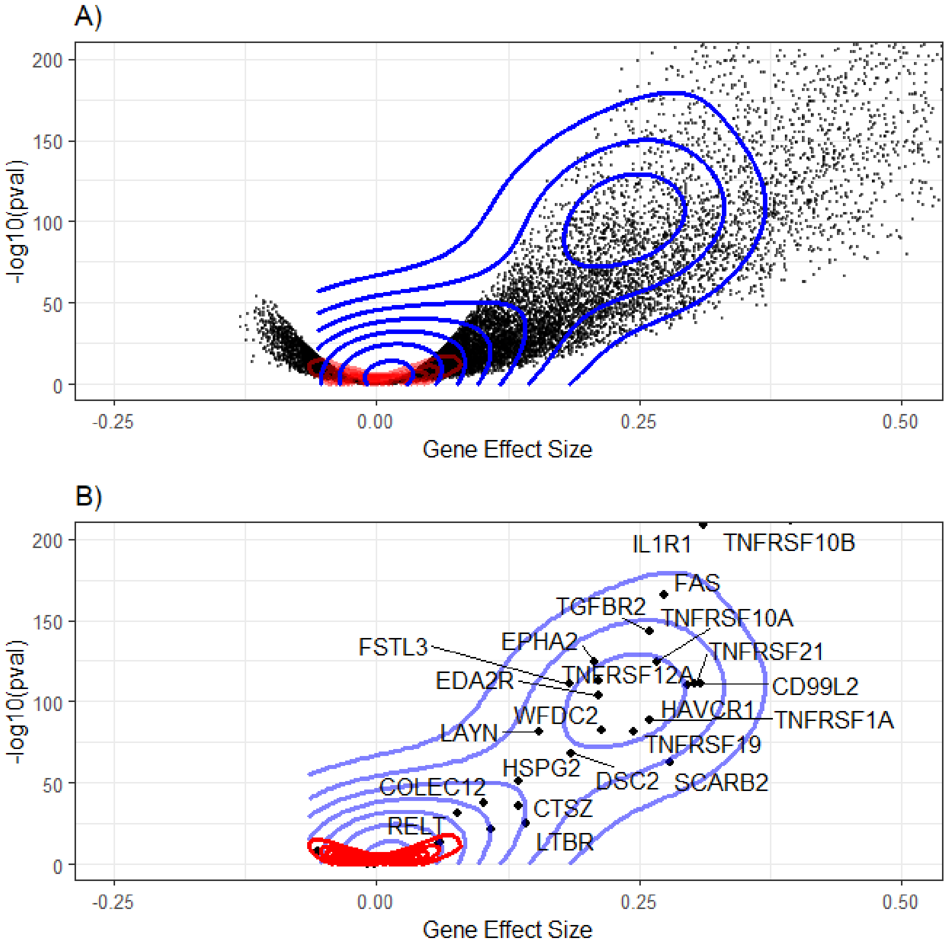

Figure S1. All protein coding genes were evaluated to determine their effect size on apoptosis index.

Panel A: Using the lmer package in R. A mixed effect per sample was included to control for non-independence of single-cell estimates for each donor. The red contours indicate the density estimate for all genes and the blue contours are the density estimate for the selected 46 circulating proteins. The axes represent the beta coefficient for expression individual genes to estimate aggregate apoptosis index with their corresponding p-values.

Panel B: The subset of 46 circulating proteins is shown with their corresponding effect size estimates. The red contours indicate the density estimate for all genes and the blue contours are the density estimate for the selected 46 circulating proteins.

A)

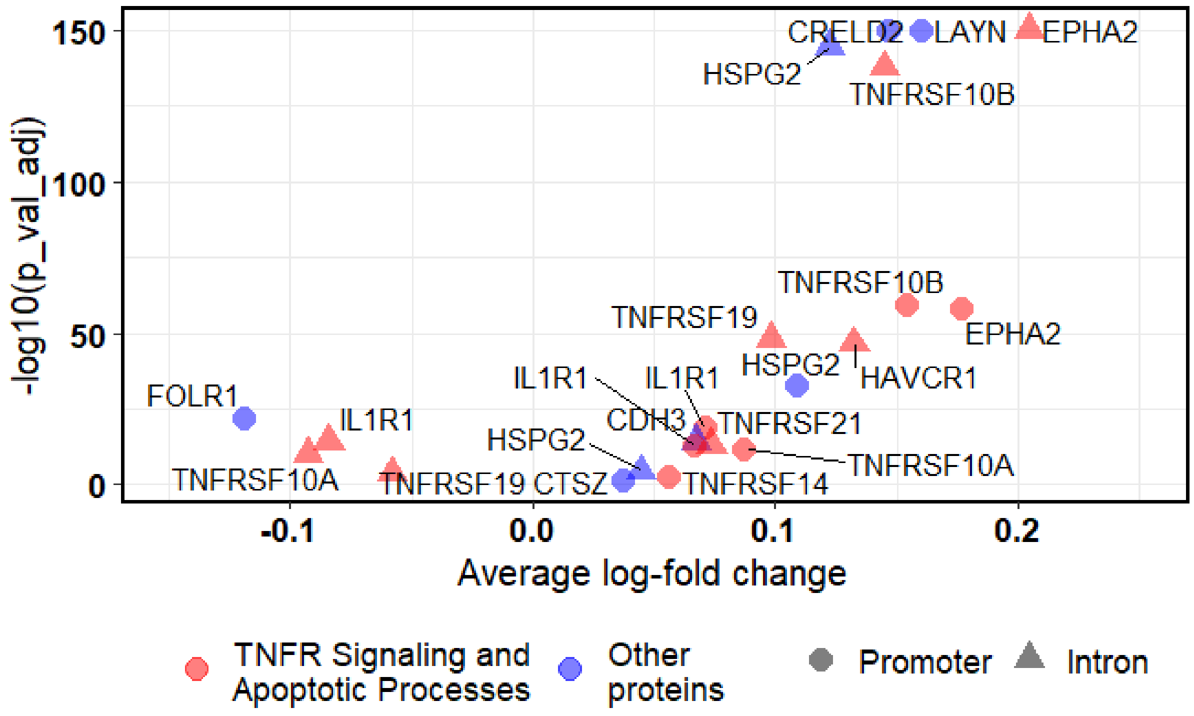

Figure S2: Differential chromatin accessibility of genes encoding for 46 circulating ESKD associated proteins in PT_VCAM1 vs. control proximal tubule cells.

Only genes that met the Benjamini-Hochberg adjusted p-value threshold (p <0.05) are displayed. Red circles indicate apoptosis/TNF receptors proteins and blue circle indicate other proteins.

TNFR: tumor necrosis factor receptor. ESKD: end-stage kidney disease.

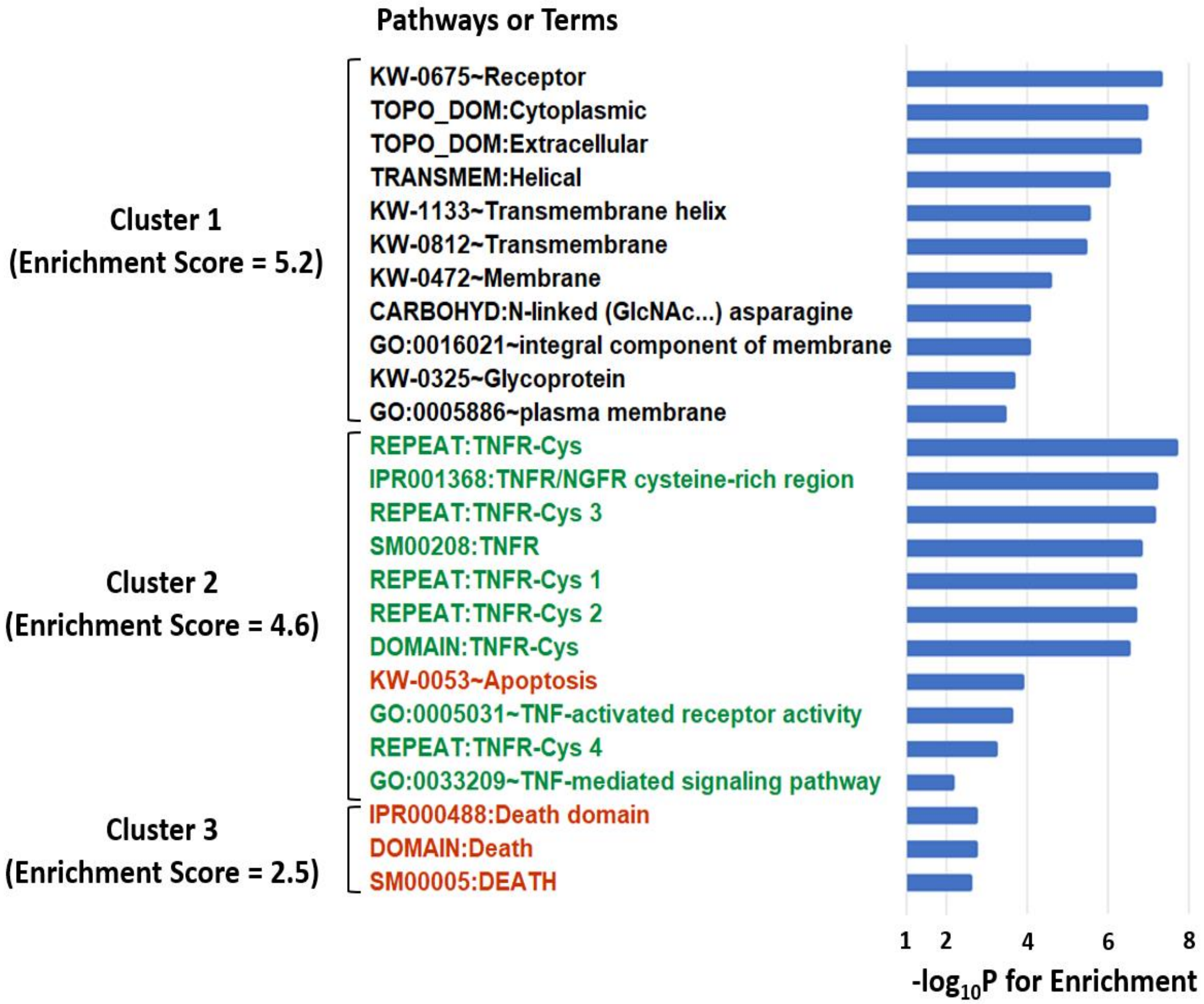

Figure S3. Pathway analysis for SOMAscan results. Reanalyzed data from our previous publication (S5).

Clusters of enriched pathways by 40 end-stage kidney disease (ESKD) risk associated proteins are shown. For pathway enrichment analysis, we applied the functional annotation clustering in DAVID software (version 6.8). All 1,129 proteins included in the SOMAscan platform were used as background for the analysis. The geometric mean (in -log scale) of the Expression Analysis Systemic Explorer (EASE) Scores (modified Fisher Exact p-values) were used to rank their biological significance. Three clusters of pathways or terms with EASE scores of $p < 0.01$ showed statistically significant enrichment.
